# Brain bases of English morphological processing: A comparison between Chinese‐English, Spanish‐English bilingual, and English monolingual children

**DOI:** 10.1111/desc.13251

**Published:** 2022-03-01

**Authors:** Xin Sun, Rebecca A. Marks, Kehui Zhang, Chi‐Lin Yu, Rachel L. Eggleston, Nia Nickerson, Tai‐Li Chou, Xiao‐Su Hu, Twila Tardif, Teresa Satterfield, Ioulia Kovelman

**Affiliations:** ^1^ Department of Psychology University of Michigan Ann Arbor Ann Arbor Michigan USA; ^2^ Department of Psychology National Taiwan University Taipei Taiwan

**Keywords:** bilingualism, brain development, Chinese‐English bilingual, fNIRS, morphological processing, Spanish‐English bilingual

## Abstract

How do early bilingual experiences influence children's neural architecture for word processing? Dual language acquisition can yield *common* influences that may be shared across different bilingual groups, as well as *language‐specific* influences stemming from a given language pairing. To investigate these effects, we examined bilingual English speakers of Chinese or Spanish, and English monolinguals, all raised in the US (*N *= 152, ages 5–10). Children completed an English morphological word processing task during fNIRS neuroimaging. The findings revealed both language‐specific and shared bilingual effects. The language‐specific effects were that Chinese and Spanish bilinguals showed principled differences in their neural organization for English lexical morphology. The common bilingual effects shared by the two groups were that in both bilingual groups, increased home language proficiency was associated with stronger left superior temporal gyrus (STG) activation when processing the English word structures that are most *dissimilar* from the home language. The findings inform theories of language and brain development during the key periods of neural reorganization for learning to read by illuminating experience‐based plasticity in linguistically diverse learners.

## 1 INTRODUCTION

Language scientists have long sought to understand how two languages interact in the bilingual brain. When a child acquires two languages simultaneously early in life, their brain is highly plastic and sensitive to change, and bilingualism thus offers a unique lens to understand the nature of this plasticity (Werker & Hensch, 2015). Dual language acquisition can yield *commonalities* on language processing among different bilingual groups, making it distinct from monolinguals (Abutalebi & Green, 2016; DeLuca et al., 2020; Jasinska et al., 2017; Kovelman et al., 2008). Importantly, cross‐linguistic interactions also vary as a function of the two languages that each bilingual speaks, thereby creating *language‐specific* transfer effects (Chung et al., 2019; Ip et al., 2017). Theories of bilingualism posit that such cross‐linguistic transfer effects can have substantial impacts on children's spoken language and emerging word reading skills (Chung et al., 2019). Therefore, to shed light on mechanisms underlying bilingual effects in word processing during the key periods of neural reorganization for learning to read, the current study examined spoken word processing in Spanish‐English and Chinese‐English bilinguals as well as monolingual English children.

### 1.1 Bilingualism changes the mind and brain

Bilingual experience changes the mind and brain. In particular, research has suggested that a *neural signature of bilingualism* may distinguish the bilingual and monolingual neural organization, associated with the added computational challenge of acquiring two languages from early life (DeLuca et al., 2020; Jasinska & Petitto, 2013, 2014; Kovelman et al., 2008). Adult bilingual neuroimaging research suggests powerful transfer effects that influence bilingual adults’ processing of their new languages (see a comprehensive summary by H. Liu & Cao, 2016). However, many factors, such as the age of acquisition and language proficiency, can overshadow the subtle cross‐linguistic effects. For example, second language processing often adds to the overall cognitive load of language processing, making it difficult to disentangle the added cognitive load from cross‐linguistic interactions (H. Liu & Cao, 2016).

In contrast to adult second language learners, children with early bilingual exposure acquire their two languages during the key periods of brain development for language with maximal sensitivity to linguistic input (Werker & Hensch, 2015). Early bilingual exposure typically yields balanced proficiency and simultaneous neural development for the two languages. Early simultaneous bilinguals may thus build unique associations between their language proficiency and neural architecture for word processing (Kasparian et al., 2017). Therefore, an examination into early dual first language children can provide meaningful evidence in the understanding of the bilingual brain (Kovelman et al., 2008; Petitto & Kovelman, 2003). In the present study, we use lexical morphological processing as a lens to examine the effects of bilingual experience on children's neural architecture for language.

### 1.2 Bilingual transfer effects on lexical morphological processing

Words are universally composed of one or more units of meaning, called *morphemes*. Recognizing these meaningful units of words (e.g., *dish‐wash‐er, friend‐li‐est*) requires familiarity with lexical units and the specific rules by which these units combine to form words in a given language (Ke et al., 2021; Sun et al., 2021). Critically, these rules vary across languages. For instance, many languages allow for lexical roots to be joined together to form novel compound words. In English, compound words are typically right‐headed (e.g., *dishwasher* ), whereas Spanish compounds are typically left‐headed (e.g., *lavaplatos*, or wash‐plates). As a more common feature of word construction, over 90% of the Chinese words are lexical compounds and they are typically right‐headed (e.g., 洗碗机, or wash‐bowl‐machine). An inquiry into lexical morphological awareness thus offers a unique lens into bilingual effects on children's emerging neural architecture for language, because English, Spanish, and Chinese all feature morphology, but in different ways, which could help shine light on the underlying mechanisms by which young learners recognize complex polysyllabic lexical items. To inform theories of bilingual language development during key periods of learning to read, the present study aimed to examine the effects of bilingual experiences with either Chinese or Spanish on the neuro‐cognitive mechanisms of English lexical morphology processing in the two bilingual groups with comparable English proficiency as compared to each other, as well as in comparison to reading‐proficiency matched English monolinguals.

**RESEARCH HIGHLIGHTS**

- This study investigates the effects of early bilingualism on children's neural architecture for word processing by comparing monolinguals and bilinguals with a lexical morphology task
- Language‐specific transfer effects revealed principled functional activation differences between Chinese‐English bilinguals, Spanish‐English bilinguals, and English monolinguals
- Common to both bilingual groups, home language proficiency was positively associated with left STG activation when processing English words that have morphological structures most distinct from their home language
- Successful dual first‐language acquisition is likely made possible by automating linguistically shared processes and heightened sensitivity to dissimilar processes

According to the Interactive Transfer Framework (Chung et al., 2019), the transfer of children's emerging language and literacy skills typically happens at points of similarity between two languages, points that include both spoken and orthographic word recognition processes. Of note here is that whereas spoken language proficiency precedes and predicts learning to read, starting in elementary school years, gains in children's spoken language competence are often intertwined with their gains in orthographic experiences and proficiency. In the case of lexical morphology, there are many shared components in English and Spanish, including cognates (e.g., *communicate/comunicar*) and derivational morphemes (e.g., *‐al*). In these languages, derived words with a single root morpheme and one or more affixes (e.g., *un‐communicat‐ive*) occur with high frequency. These shared properties yield meaningful morphological transfer in Spanish‐English bilinguals through the high degree of correlation between morphological skills across their two languages (Ramírez et al., 2010). Intriguingly, exposure to Spanish, a language where derivational morphological constructions are more frequent, productive, and expressed through transparent sound‐to‐print mappings that often correspond to those found in English, may even boost morphological development in proficient English speakers (Kuo et al., 2017). Yet, it remains generally unknown whether dual first language Spanish‐English experience may yield transfer effects in the neural functionality of morphological processing for children whose primary language of literacy instruction is English.

In contrast to Spanish and English, most Chinese words are morphological compounds that are constructed from combining root morphemes (e.g., *snow‐man*), and very few are derivationally constructed. Of note is that in print, Chinese characters typically correspond to root morphemes. Research thus finds a stronger interrelation between compound awareness and early literacy development and dyslexia in Chinese relative to English (Tong et al., 2017). These cross‐linguistic differences help explain the observation that young Chinese‐English bilinguals exhibit strong compounding but not much of a derivational morphology transfer effect (Luo et al., 2014; Pasquarella et al., 2011; M. Wang et al., 2006). For instance, in Ramírez et al. (2011), Chinese‐English bilinguals performed comparably to English monolinguals, and better than Spanish‐English bilinguals on compound awareness; however, on derivational awareness, they demonstrated lower proficiency than the other groups. The transfer of compounding between Chinese and English is particularly important to note, as it suggests the transfer of the morphological awareness principles despite little to no lexical overlap across languages.

In sum, morphological awareness reflects language‐specific properties, and behavioral evidence suggests robust transfer effects at points of structural similarity between the two languages. Moreover, the direction of transfer typically occurs from the language which has frequent or salient morphological features such as derivational morphology in Spanish and compound morphology in Chinese (Chung et al., 2019) to the less morphologically rich language. Therefore, a comparison between Spanish‐English and Chinese‐English bilinguals may lead to a principled approach of uncovering the effects of cross‐linguistic bilingual experiences on children's neural organization for language.

### 1.3 Brain bases of morphological word processing

Adult neuroimaging research on lexical morphology typically addresses the core questions about mental processes engaged in word recognition and analyses, thereby offering a framework to yield our predictions for bilingual development. For instance, the neurocognitive framework offered by Gwilliams (2020) poses a multi‐stage process for recognizing a morphologically constructed word. The process includes morpheme identification, lexical access, and morphological composition. Each of the stages is supported by different but interconnected neural mechanisms. Morphemic identification involves segmenting words into morpho‐syllabic constituents and engages regions such as the left superior temporal gyrus (STG), known for its role in phonological segmentation (Ettinger et al., 2014). Lexical access involves matching the segmented morphological forms with their respective meanings and engages regions such as the left middle temporal gyrus (MTG), known for its role in semantic processing (Binder, 2017). As *free root morphemes* often have greater lexical transparency (e.g., *smart, friend*), the process may stop at this point. For example, lexical judgment tasks with English compound words (vs. nonwords) revealed lexical retrieval processes reflected by left MTG engagement (Fiorentino & Poeppel, 2007). In contrast, *derivational affixes* are more abstract and analytically demanding (e.g., *‐est, ‐ly*), thus more likely to incur higher‐order analytical steps and engage the left inferior frontal gyrus (IFG), known for its role in complex structural and phonemic analyses of language as well as complex sound‐to‐meaning integration (Enge et al., 2020). Indeed, lexical decision tasks with derived words (i.e., agree‐able) elicited stronger left IFG and STG activations than simple single‐morpheme words (Vannest et al., 2011).

These adult neuroimaging findings for morphological processing are a good fit for the dual‐stream framework of language processing (Hickock & Poeppel, 2007). The dual‐stream framework poses two interrelated but specialized neural pathways for phonological and lexico‐semantic processes. The dorsal stream includes posterior inferior and superior temporal gyri (dIFG, STG) and helps support phonological processes. The ventral stream includes the ventral inferior and middle temporal gyri (vIFG, MTG) and helps support the semantic analyses, with STG and IFG regions also supporting the integration of lexico‐semantic and morpho‐phonological processes. In proficient adult speakers, the relative engagement of these systems during a lexical morphology task likely varies as a function of cognitive task demands, such as compound versus derived morphology (Devlin et al., 2004; Kirby & Bowers, 2017).

Neurodevelopmental research into lexical morphology similarly reveals distinct processing systems across young children with varied language experiences and (dis)abilities. This work has often been framed in terms of early literacy acquisition and dyslexia. In English, derivational morphology has been closely linked to successful literacy development as well as reading impairment (Tong et al., 2011; Tong, Deacon, et al., 2014). In keeping with this behavioral evidence, English‐speaking children with dyslexia exhibited reduced activation compared to their typical reading peers in brain regions associated with phonological and orthographic processes (left frontal and temporal‐occipital areas) during a visual morphology task (Aylward et al., 2003). In Finnish, a derivationally‐rich language, listening to sentences with correctly and incorrectly constructed derived words incurred robust activations along the dorsal/phonological network (left IFG and STG; Louleli et al., 2020). In contrast to English and Finnish, compound morphology is closely associated with literacy success in Chinese (P. D. Liu & McBride‐Chang, 2010). Typically developing Chinese readers, but not their peers with dyslexia, demonstrated a semantic ERP element (N400) during a lexical decision task with compound words and non‐words (Tong, Chung, et al., 2014). Altogether, child findings suggest that across languages, reading success is associated with the engagement of neurocognitive systems that reflect the morphological structure of a given language, including the dorsal/phonological network for derivational analysis in English/Finnish and ventral/semantic network for lexical retrieval in Chinese.

Neuroimaging studies with bilingual children and adults often echo the developmental work that uncovers the interplay between two languages in bilinguals’ literacy development. These studies often have a strong focus on the role of phonology‐to‐orthography transparency on cross‐linguistic transfer. For instance, bilingual children with Hindi L1 (phonologically transparent) and English L2 (phonologically opaque) showed stronger engagement of the phonological networks when reading in English as compared to monolingual English speakers (Das et al., 2011), whereas the opposite is true for those whose first language is even less phonologically transparent than English such as Chinese (H. Liu & Cao, 2016). The question we ask here is whether such a bilingual transfer effect is also possible for spoken word recognition and processing. One prior study with young Chinese‐English bilinguals raised in the US offers encouraging results (Ip et al., 2017). The study asked young Chinese‐English bilinguals and English monolinguals to complete a derivational morphology task in English and a compound morphology task in Chinese. In English, the children heard pairs of words and decided if the second word, a novel or otherwise low‐frequency derived word, was acceptable or not (yes for *jump*, *re‐jump*; no for *cow*, *re‐cow*). In Chinese, children also heard pairs of words and decided if the second word, a novel or low‐frequency compound word was acceptable or not. The results revealed that whereas across groups/languages, participants engaged both phonological and lexico‐semantic networks, the left MTG activation was more significant in bilinguals across both languages compared to English monolinguals. Left MTG functionality has been previously associated with morphological compounding and lexical root‐extraction tasks that engage the more automated lexical retrieval and sound‐to‐meaning integration processes (Gwilliams, 2020).

Building upon these prior bilingual works, coupled with neurodevelopmental findings and the adult neurocognitive model, we predicted that bilingual experiences with lexical compounding in Chinese might be associated with more automated lexico‐semantic retrieval processes of the ventral network, especially the MTG region and especially during the lexical compound task. In contrast, bilingual experiences with Spanish might be associated with the phonological or dorsal network, especially the left frontal regions critical to processing the more semantically‐abstract and analytically‐complex derivational affixes.

### 1.4 The present study

In the present study, we examined how early bilingual experiences with structurally different languages, Chinese or Spanish, might be associated with children's emerging neural architecture for morphological processing in English. According to the Unified Bilingual Experience Trajectory Model (UBET, DeLuca et al., 2020), bilingual experiences alter the neural functionality of language processing, yielding *shared* bilingual impacts. Guided by this model, we hypothesize that there may be general neural patterns associated with bilingual experiences in either Chinese or Spanish. Moreover, according to the Interactive Transfer Framework (Chung et al., 2019), bilinguals demonstrate *language‐specific* transfer on elements that are shared across their two languages. Guided by this framework, we hypothesize that bilingual experiences with Chinese, a language characterized by compound morphology, should influence how children process English word roots and compounds. In contrast, bilingual experience with Spanish, a language with productive derivational structures, should influence how children process English derivational affixes. Specifically, we predicted that (1) Chinese‐English bilingual children may demonstrate stronger automaticity in processing English word roots/compounds, reflected through the ventral network (i.e., MTG) and less automaticity for derivational structures, reflected through the dorsal network (i.e., dIFG/STG); and (2) in contrast, Spanish‐English bilingual children may demonstrate stronger automaticity in processing English derivational structures (i.e., dorsal networks, dIFG/STG). In addition, we also explored cross‐linguistic bilingual transfer effects through brain‐behavioral correlation analyses between bilingual children's heritage language proficiency and neural activations of English morphology. As the brain‐behavioral association transfer was exploratory, there were no specific hypotheses, but we expected the associations to exist in key regions for morphological processing (i.e., left IFG, STG, and/or MTG).

To test these predictions, we asked Chinese‐English bilingual and Spanish‐English bilingual children with early and systematic exposure to two languages, as well as English monolingual children to complete a lexical morphology task during functional Near‐Infrared Spectroscopy (fNIRS) neuroimaging. The morphology task included an experimental compound morphology condition, an experimental derivational morphology condition, and a control word recognition condition. They also completed behavioral language assessments in each of their languages.

## 2 METHOD

### 2.1 Participants

Participants were drawn from a larger project with children who had typical and delayed reading proficiency. All children attended English‐only schools in southeast Michigan, USA. We used the following inclusion criteria. First, monolingual participants had exposure to English from birth, with no systematic exposure to other languages. Bilingual participants had received systematic exposure to their home language at birth. Second, all participants had typical English oral language proficiency, as indicated by a standard English vocabulary score of 85 or greater (Peabody Picture Vocabulary Test 5, PPVT‐5, D. M. Dunn, 2019). Third, bilingual participants all had early exposure to English: they attended English‐only schools at or prior to kindergarten. Both bilingual groups on average started saying English words between 1.5 and 2.5 years, English sentences between 2 and 3 years, and they showed no group difference. The age of the first English word and the first English sentence were measured by questions “At what age did your child say his/her first English word?” and “At what age did your child say his/her first English sentence?” (Table 1).

**TABLE 1:** Behavioral and neuroimaging task performance (Ms and SDs) by language group

	Chinese bilingual	Spanish bilingual	English monolingual		
	*M(SD)*	*M(SD)*	*M(SD)*	*F*‐Value	*p*‐Value
*N*	48	50	54	–	–
Age	7.63 (1.44)	7.84 (1.22)	7.66 (1.32)	0.01	0.921
English age of acquisition (word)^a^	3.09 (1.67)	3.19 (1.92)	–	0.06	0.803
English age of acquisition (sentence)^a^	4.60 (1.48)	4.60 (1.57)	–	<0.01	0.989
*English raw score*					
Vocabulary	145.09 (32.95)	144.30 (28.14)	158.40 (26.84)	5.34	0.022
Word reading	50.47 (14.36)	48.54 (15.27)	46.75 (16.79)	1.43	0.234
Reading comprehension	27.22 (8.35)	24.64 (6.88)	26.12 (9.18)	0.37	0.540
Morphological awareness	24.54 (11.18)	24.42 (10.01)	25.31 (11.32)	0.14	0.712
Phonological awareness	22.63 (7.48)	23.62 (7.23)	21.42 (7.79)	0.71	0.401
*English standard score*					
Vocabulary^b^	106.34 (18.06)	102.22 (17.75)	117.15 (18.64)	9.03	0.003
Word reading^b^	116.64 (14.91)	111.46 (18.99)	111.43 (16.46)	3.50	0.064
Reading comprehension^b^	109.54 (13.19)	101.81 (11.41)	105.15 (12.34)	1.78	0.185
Phonological awareness^c^	11.15 (2.77)	11.37 (3.08)	10.48 (2.49)	1.48	0.225
*Heritage language (Spanish/Chinese)*					
Vocabulary raw score	56.07 (16.42)	67.74 (19.44)	–	–	–
Vocabulary standard score	91.07 (17.58)	108.38 (18.12)	–	–	–
*In‐scanner task accuracy (% correct)*					
Roots/compounds condition	83.59 (14.39)	78.75 (14.78)	82.78 (13.02)	0.06	0.814
Affixes/derivations condition	62.63 (16.49)	57.50 (15.78)	61.32 (13.82)	0.14	0.706
Word recognition control	95.83 (6.23)	96.00 (7.12)	94.69 (7.39)	0.71	0.401

^a^English Age of Acquisition was measured by two items probing children's age of first word and sentence, respectively: “At what age did your child say his/her first English word/sentence?” They used a six‐point scale, 1 = 9–11 months, 2 = 1–1.5 years, 3 = 1.5–2 years, 4 = 2–2.5 years, 5 = 2.5–3 years, 6 = older than 3 years.

^b^Standard *M*(*SD*) = 100 (15).

^c^Standard *M* = 10 (8–12 fall into a typical range).

Motivated by our interest in the effects of early bilingual experiences and to avoid the confounding effects of proficiency, we included bilingual children with early and systematic bilingual experiences, and purposefully matched the three groups based on their English literacy proficiency. Our matching criteria were principled because lexical morphology development is often studied in the context of literacy acquisition and it has reciprocal relationship with reading growth (Carlisle, 1995; Chung et al., 2019). English vocabulary was not a matching criterion as bilingual children generally had lower English vocabulary than monolinguals due to shared vocabulary by their two languages (Poulin‐Dubois et al., 2013).

The final sample included *N* = 152 children (75 girls), *M*(*SD*)_Age_ = 7.71(1.32), including 48 Chinese‐English bilinguals (23 girls); 50 Spanish‐English bilinguals (22 girls); and 54 English monolingual children (30 girls). There were no age or gender distribution differences across groups (all *p* > 0.05). All participants were typically developing children with no history of hearing, cognitive, or neurological impairments. The study was approved by the institutional review boards for research with human participants.

### 2.2 Measures and procedure

All participants completed standardized and experimenter‐made English language and literacy assessments, including phonological awareness (Elision subtest, Comprehensive Test of Phonological Processing, CTOPP; Wagner et al., 1999), Single‐word reading (Letter‐word Identification subtest, Woodcock‐Johnson IV, WJ‐IV; Schrank et al., 2014), Reading Comprehension (Passage Comprehension subtest, Woodcock‐Johnson IV, WJ‐IV; Schrank et al., 2014), and lexical morphological awareness (Early Lexical Morphology Measure, ELMM; Marks, Eggleston, et al., 2021). Bilingual participants also completed an assessment of Spanish or Chinese vocabulary (Chinese: Peabody Picture Vocabulary Test‐Revised, PPVT‐R, Lu & Liu, 1998; Spanish: Test de Vocabulario en Imágenes Peabody, TVIP, L. Dunn et al., 1986).

Each participant completed behavioral and neuroimaging sessions in one visit. All tasks were administered by native speakers of each language. Table 1 shows participants’ performance on all behavioral and neuroimaging tasks.

### 2.3 Neuroimaging morphological awareness task

The neuroimaging morphological awareness task was an auditory task with three conditions: Free Roots/Compounds, Affixes/Derivations, and Word Recognition Control. For all three conditions, each trial included three words: a target word and two comparison words. All stimuli can be found in Supplement Table S1.

#### 2.3.1 Free roots/compounds experimental condition

In the Free Roots/Compounds condition, the correct answer shares a morphemic root with the target/first word, and the incorrect answer is a phonological distractor. For example, in the trial *bedroom, classroom, mushroom*, the correct answer is *classroom*, as it shares a root morpheme with the compound word *bedroom*, whereas the phonological distractor *mushroom* is an incorrect choice. This condition also included items that shared the same root morpheme but were not compound words, for example, *winner, winning, window*.

#### 2.3.2 Affixes/derivations experimental condition

In the Derivational Affixes condition, the correct answer shares a derivational affix with the target/first word, and the incorrect answer is again a phonological distractor. For example, in the trial *disagree*, *dishonest, distance*, the word *dishonest* matches *disagree* with the same prefix, and *distance* is a phonological distractor. Within this condition, eight trials match on Latinate affixes (e.g., *dis‐, ‐ment*) and eight trials match on Germanic or middle/old English affixes (e.g., *mis‐, ‐est*). Note that the two experimental conditions asked participants to match different types of morphemes: the free roots/compounds condition focused on *word root* match, whereas the affixes/derivations condition focused on *affix* match.

#### 2.3.3 Word recognition control condition

Finally, the control Word Recognition condition tests children's whole word processing and it matches the whole word instead of shared morphemes (e.g., *alarm* – *alarm* – *marker*).

### 2.4 Neuroimaging task procedure and stimuli

**FIGURE 1:**
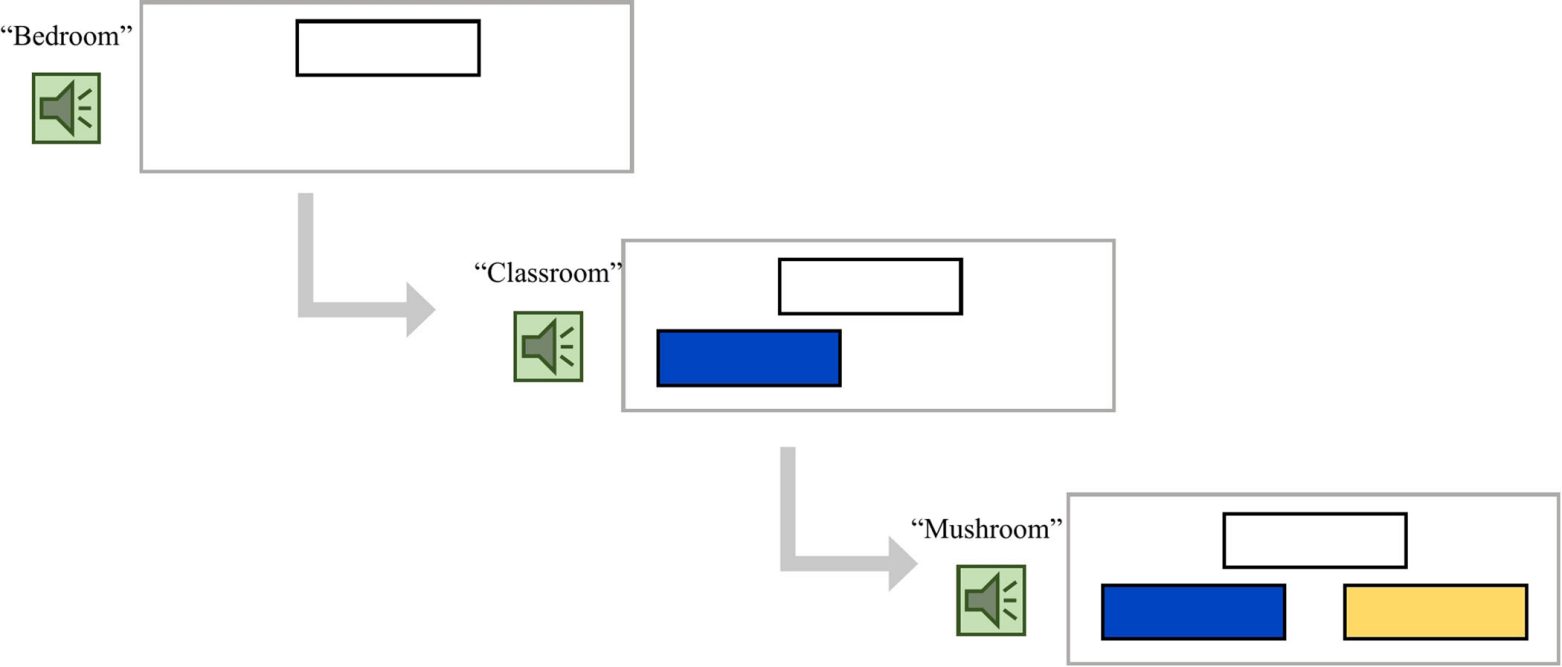
An example trial of the fNIRS morphological processing task note. For each trial, participants first hear the target word (e.g., “bedroom”) and see a white box on the top of the screen, then they hear two words of choices (e.g., “classroom,” “mushroom”) and simultaneously see a blue and a yellow box, respectively

A practice session was conducted before the actual task to familiarize children with the task. For the first three trials, children listened to three words and were presented with three pictures in a booklet (first/target picture on the top, e.g., *classroom*, second and third pictures of choices on the bottom left and right, e.g., *bedroom* and *mushroom*). They were asked to pick whether the second or third word matched the first word by pointing to the picture. Incorrect responses were discussed with the experimenter if needed to ensure that the child understood the task. Next, the experimenter directed children to a computer and asked them to complete three more trials (with pictures) but pick the word by pressing keys. Finally, the child was directed to complete three more trials without pictures (identical to the actual task as shown in Figure 1 though with different stimuli). Children were corrected with explicit explanations when they made incorrect choices and only allowed to proceed to the actual task upon a full understanding of the task procedure.

The task followed a block design and included four blocks per condition and four trials per block, adding up to 16 trials per condition, and a total of 48 trials. Each block lasted 30 s and there was a 6‐s rest in between each experimental block, adding up to ∼7.2 min. During each trial, as the first word was presented, a blank rectangle appeared on the top middle of the computer screen. Next, as the second and third words were presented, a blue and a yellow rectangle were shown on the bottom left and right of the screen, respectively (see Figure 1 for an example trial screen). Children were asked to use a button box to indicate their answers. The order of the blocks and the order of items within the blocks were randomized once (see Supplement Table S1 for the item and block sequence).

Across conditions, words were matched in the number of letters, number of phonemes, number of syllables, and age of acquisition according to the Auditory English Lexicon Project database (https://inetapps.nus.edu.sg/aelp/; Goh et al., 2020). On average, the words had *M*(*SD*) = 6.65 (1.37) letters, *M*(*SD*) = 5.63 (1.20) phonemes, and *M*(*SD*) = 2.07 (.40) syllables. Words were acquired at an average of *M*(*SD*) = 5.56 (1.45) years old. One‐way ANOVAs between words of the three conditions revealed no significant differences in any of the parameters above (all *p*s > 0.05). Words were also matched on word frequency across conditions based on the most recent version of the Corpus of Contemporary American English (COCA, https://www.english‐corpora.org/coca/, Davies, 2020).

### 2.5 fNIRS data acquisition

**FIGURE 2:**
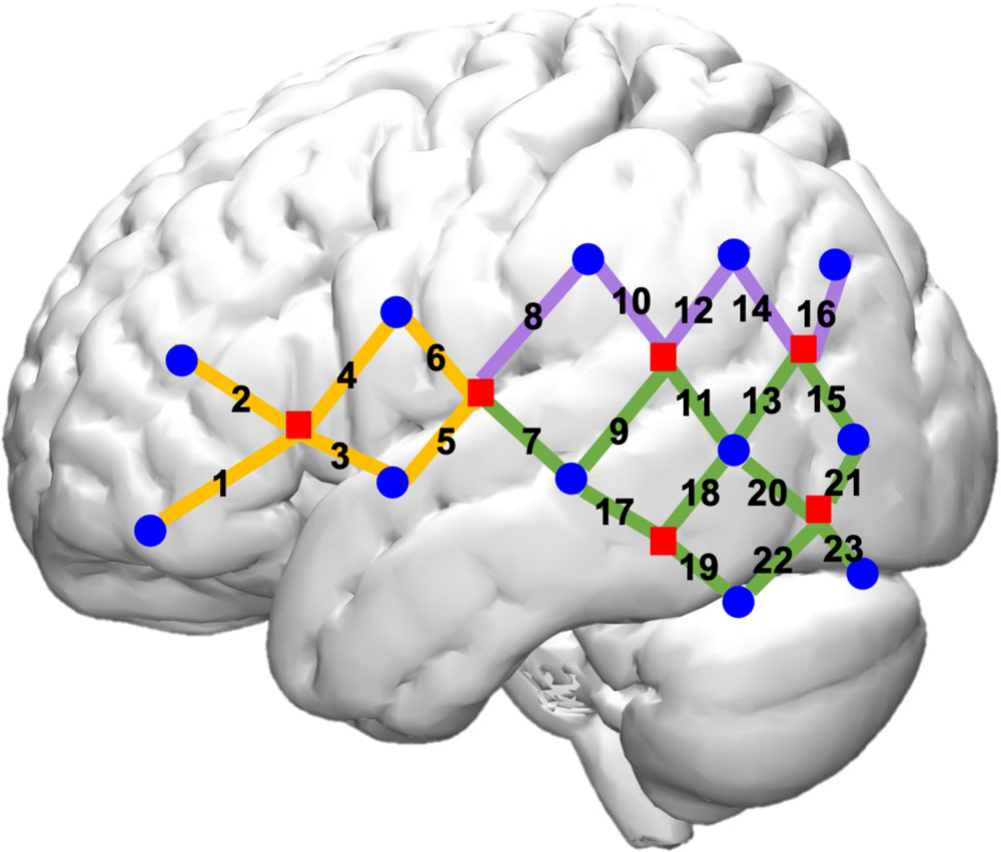
fNIRS probe setup Note. Dot shape. Square: light sources; round: light detectors. Channel color. Orange: frontal lobe; purple: parietal lobe; green: temporal lobe

The fNIRS cap was designed to cover major language and literacy brain networks as documented in prior research, including the ventral and dorsal inferior frontal, and superior and middle temporal regions. The fNIRS cap had 12 near‐infrared light emitters and 24 detectors spaced approximately 2.7 cm apart, symmetrically located on the left and right hemispheres. These optodes yielded 46 data channels (23 per hemisphere, see Figure 2). The channels broadly cover areas of language processing, including frontal, temporal, and parietal regions.

To visualize the brain regions covered by the channels (i.e., source‐detector pairs), we registered the fNIRS optodes (i.e., light sources and detectors) with a 3D digitizer and approximated the MNI coordinates of the mid‐points of each channel. Then, a rendering circle centered around each channel midpoint was drawn with a radius of 1 cm. The use of a 1 cm radius took out superficial brain layers and best captured accurate coverage of the brain regions measured by the channel (Rupawala et al., 2018). The brain areas distributed along the circle were identified as the regions covered by each channel. Specific MNI coordinates of the channel mid‐points as well as the identified brain regions for each channel was documented in Hu et al. (2020) and Marks, Labotka, et al. (2021). This approach of fNIRS channel visualization was also used in previous literature (Arredondo et al., 2017).

Trained experimenters followed standardized protocols to apply the fNIRS cap to ensure consistency across participants. Experimenters first measured participants’ nasion, inion, Fpz, left and right pre‐auricular points, and head circumference. Next, F7, F8, T3, and T4 were anchored to a specific source or detector.

fNIRS data were acquired using the TechEN‐CW6 system with 690 and 830 nm wavelengths with a 50 Hz sampling frequency. The TechCN‐CW6 software set the minimum and maximum signal‐to‐noise ratio to 80 and 120 dB, respectively. Before participants began the task, experimenters completed data quality control by checking participants’ cardiac signals across key channels of interest and confirming that the fNIRS signals among these channels were within the signal‐to‐noise range.

### 2.6 fNIRS data processing and statistical analyses

fNIRS data were analyzed with the NIRS Brain AnalyzIR, a Matlab‐based data analysis toolbox (Santosa et al., 2018), and experimenter‐developed scripts.

#### 2.6.1 Subject‐level analysis

Raw data were first trimmed to keep 5 s of pre‐ and post‐experimental task data as a baseline. Next, data were resampled from 50 to 2 Hz given that the fNIRS signal of interest lies in frequency bands of 0–1 Hz. Then, optical density data was converted to hemoglobin concentration data by applying the modified Beer‐Lambert law. Hemoglobin concentration data was then analyzed using the general linear model (GLM; Friston et al., 2007). Motion corrections were performed with an autoregressive‐whitened robust regression solution as described in Barker et al. (2013). We used the canonical hemodynamic response peaking 6‐s after trial onset as the basis function for the modeling process (Friston et al., 2007). This process produced individual‐level regression coefficients (beta values for different conditions) for HbO (oxygenated hemoglobin) and HbR (deoxygenated hemoglobin) signals collected from each channel.

#### 2.6.2 Group‐level analysis

Group‐level analyses were performed using linear mixed‐effects (LME) models for each channel. To examine the neural basis of morphological awareness of the three groups, we fitted the first LME and modeled the interaction between task condition (Root/Compound, Derivation, and Control) and participant language group (Chinese‐English, Spanish‐English bilingual, and English monolingual) to predict the individual‐level beta values (for HbO and HbR). The corresponding analytical formula is “beta ∼ group*condition + (1|Subject).” To test how home language proficiency contributes to bilinguals’ neural activity, we fitted two LMEs and used home language vocabulary to predict the neural activity for each bilingual group while controlling for the English vocabulary. The corresponding analytical formula is “beta ∼ condition*Home_language_Vocab + English_Vocab + (1|Subject).” Estimated group‐level effects for each channel were extracted to calculate contrasts between experimental and control conditions, each experimental condition across groups, as well as the brain‐behavioral associations. The group‐level effects (unstandardized beta values) for each contrast or association were plotted on the MNI 152 brain template using the previously digitized MNI coordinates (Hu et al., 2020). All statistical contrasts and associations yielded results with unadjusted *p*‐values and Benjamini‐Hochberg FDR‐adjusted *p*‐values which accounted for the number of task comparisons and channels (denoted as *q* below, Huppert et al., 2017; Santosa et al., 2018). The data analyses focused on the HbO signal as (1) HbO is the major contributor to the fNIRS signal (HbO 73%–79%; HbR 16%–22% according to a quantification study from Gagnon et al., 2012) and (2) studies have found that HbR signals are susceptible to noise (Hoshi, 2007; Strangman et al., 2002).

## 3 RESULTS

### 3.1 Language and literacy competence

All participants exhibited age‐ and grade‐appropriate English proficiency and reading skill, with mean standard scores ranging from 102.2 to 117.2 (Table 1). There were no significant group differences in word reading, phonological awareness, morphological awareness, and in‐scanner task performance as measured across raw scores as well as standard scores (One‐way ANOVA *F* values range 0.06–3.50, *p*‐values range 0.064–0.814, see Table 1).

Vocabulary performance revealed significant group differences in both raw (*F*(1,148) = 5.34, *p* = 0.022) and standard scores (*F*(1,148) = 9.03, *p* = 0.003). Post‐hoc pairwise comparisons (Bonferroni corrected at *p* = 0.017) revealed that monolinguals outperformed both bilingual groups in their standard scores (Spanish bilinguals: *p* < 0.001; Chinese bilinguals: *p* = 0.004), and the Spanish bilinguals in their raw scores (*p* = 0.016). Yet, the two bilingual groups did not differ from one another (raw: *p* = 0.895; standard: *p* = 0.272).

To examine whether the three groups performed similarly across the three conditions, a 3 (Group: Chinese bilingual, Spanish bilingual, English monolingual) * 3 (Condition: Free Roots/Compounds, Affixes/Derivations, Control) mixed ANOVA was conducted to predict task accuracy. Results revealed a significant main effect of Condition (*F*(2, 296) = 514.24, *p* < 0.001). Post hoc pairwise analysis showed that the control condition yielded the highest accuracy *M*(*SD*) = 95.34% (7.01%); followed by the free root/compound condition, *M*(*SD*) = 82.11% (13.67%); followed by the derivational affixes condition *M*(*SD*) = 60.47% (15.32%), all *p*s < 0.001. Moreover, both the Group main effect and the Condition*Group interaction were not significant (Group: *F*(2, 148) = 1.37, *p* = 0.257; Condition*Group: *F*(4, 296) = 1.66, *p* = 0.160).

In an additional post hoc analysis of the Affixes condition specifically, we tested for group differences in children's competence with morphemes of Latin (e.g., ‐ment) or Germanic (‐est) origin. Although there were no group differences at the condition level, it remained possible that Spanish bilinguals might show an advantage on affixes of Latin origin. Thus, we conducted a 3 (Group) *2 (Morphemic type: Latinate, Germanic or middle/old English) mixed ANOVA and again found that the Group main effect was not significant, *F*(2, 148) = 1.42, *p* = 0.245. The morphemic type main effect was significant that items with Germanic affixes (*M*(*SD*) = 63.2% (18.2%)) yielded significantly higher accuracy than items with Latinate affixes/derivations (*M*(*SD*) = 57.5% (20.1%), *F*(1, 148) = 10.04, *p* = 0.002). The interaction reached a marginal non‐significance, *F*(2, 148) = 2.58, *p* = 0.079. Post hoc analyses revealed that, for each morpheme type, task performance was not significantly different across language groups, items with Germanic affixes (*F*(2, 148) = 1.76, *p* = 0.175), and with Latinate affixes (*F*(2, 148) = 1.89, *p* = 0.154). English monolinguals performed equivalently on the Latinate (*M*(*SD*) = 60.8% (19.3%)) and Germanic items (*M*(*SD*) = 60.8% (18.0%), *p* = 1.00), in contrast, both bilingual groups performed significantly better on Germanic items (*M*(*SD*) Spanish bilinguals: 61.8% (17.8%) vs. 53.3% (20.2%), *p* = 0.007; Chinese bilinguals: 67.2% (18.7%) vs. 58.1% (20.5%), *p* = 0.005).

### 3.2 fNIRS neuroimaging results

#### 3.2.1 Morphological processing of roots and affixes in monolinguals and bilinguals

**FIGURE 3:**
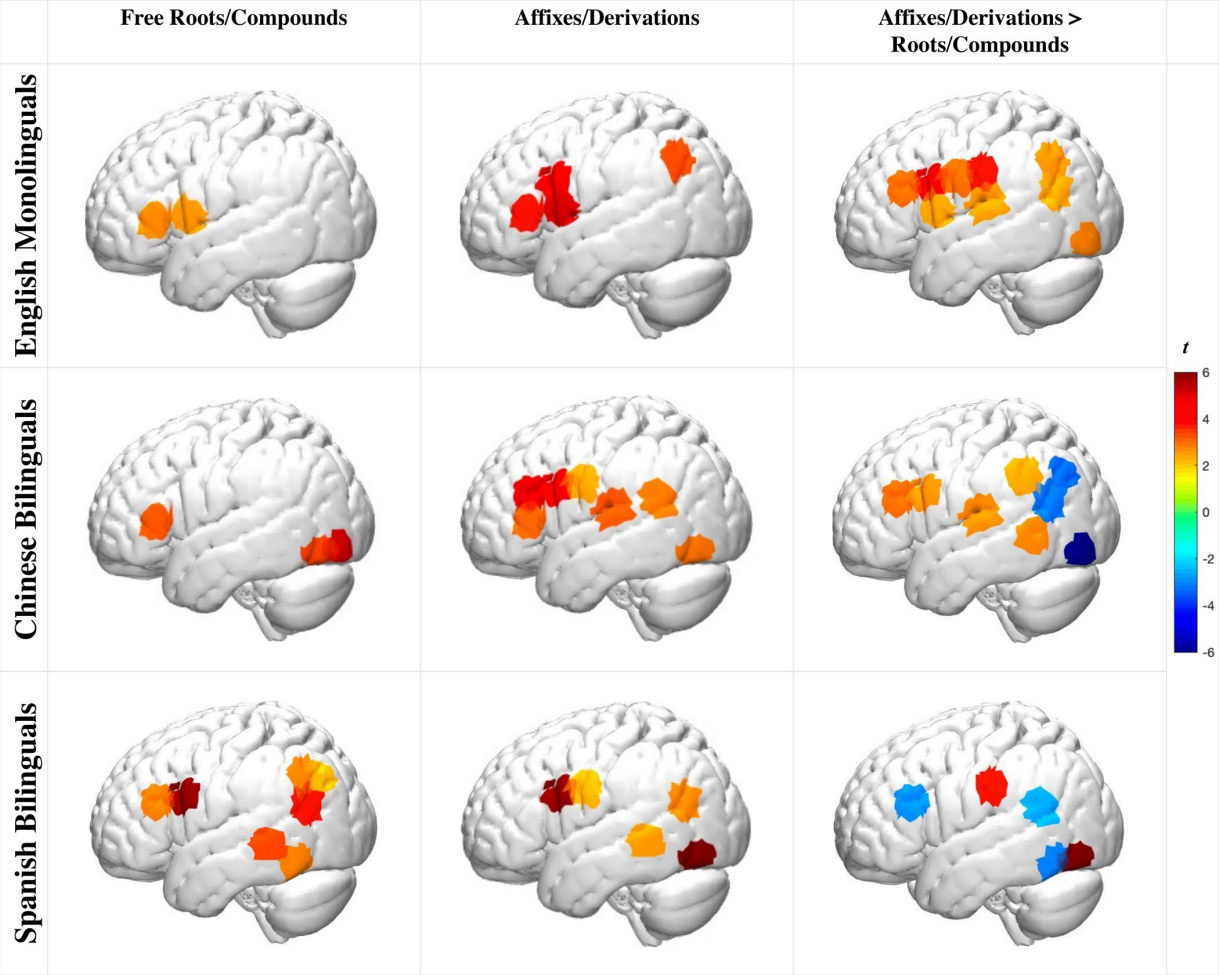
Participants’ brain activation in the language hemisphere during Free Roots/Compounds and Affixes/Derivations morphology conditions (task > control) as well as direct comparisons of the two conditions (task > rest contrasts compared; all FDR adjusted *q* < 0.05)

To investigate the neural bases of morphological awareness in children of different linguistic backgrounds, we first examined within‐group effects of each morphological condition and then the differences between the two (Figure 3 for the left hemisphere activations, for bilateral activations, see Supplementary Figure S1; detailed bilateral *beta* and *t*‐values see Supplementary Table S2, S3, S4, and S5, all FDR‐corrected *q* < 0.05).

##### Free roots/compounds

During the roots/compounds condition (e.g., *winner – winning – window*), all groups showed left frontal activation (Figure 3, Table S1; task > control contrasts). Chinese bilinguals also showed bilateral occipital and right parietal activation. Spanish bilinguals also showed left middle and inferior temporal as well as left parietal activation.

##### Affixes/derivations

During the affixes/derivations condition (e.g., *running – jumping – ceiling)*, all groups also showed left frontal activation (Figure 3, Table S3; task > control contrasts). Monolinguals showed additional activation in bilateral parietal and right postcentral regions. Chinese bilinguals showed additional activation in left temporal, right parietal, and bilateral inferior‐temporal regions. Spanish bilinguals showed additional activation in the left middle and inferior temporal and parietal regions.

##### Affixes/derivations > roots/compounds

The affixes/derivations condition elicited a stronger pattern of neural activity in English monolinguals and Chinese bilinguals, especially in the left frontal, left temporal, and bilateral parietal regions (Figure 3, Table S4 and S5; task > rest contrasts compared across the two conditions). In contrast, the Spanish bilinguals showed a different pattern, with stronger activation in the left parietal, left occipital, and right precentral regions for the affixes/derivations condition, but stronger activation in bilateral frontal, left temporal, and right parietal for the root/compound condition.

#### 3.2.2 Group differences in morphological awareness

**FIGURE 4:**
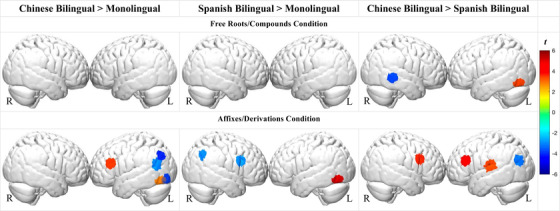
Brain activation cross‐group contrasts in the free roots/compounds and affixes/derivations conditions (all FDR adjusted *q* < 0.05)

To investigate group differences, we compared bilingual and monolingual groups across the experimental conditions (FDR corrected *q* < 0.05, Figure 4, specific *beta* and *t* values see Table S6).

##### Chinese bilinguals

For the roots/compounds condition, Chinese bilinguals exhibited greater left occipital and reduced right middle temporal activation, relative to Spanish bilinguals. There were no differences between Chinese bilinguals and monolinguals during the roots/compounds condition. For the affixes/derivations condition, Chinese bilinguals exhibited stronger left inferior/middle frontal but lower left parietal activation relative to the other two groups. Relative to Spanish bilinguals, Chinese bilinguals also showed stronger activation in the left superior temporal region.

##### Spanish bilinguals

For the roots/compounds condition, there was no significance between Spanish bilinguals and English monolinguals. For the affixes/derivations condition, Spanish bilinguals exhibited stronger left occipital but lower right frontal and right parietal activations relative to monolinguals.

#### 3.2.3 Exploring bilingual proficiency effects

**FIGURE 5:**
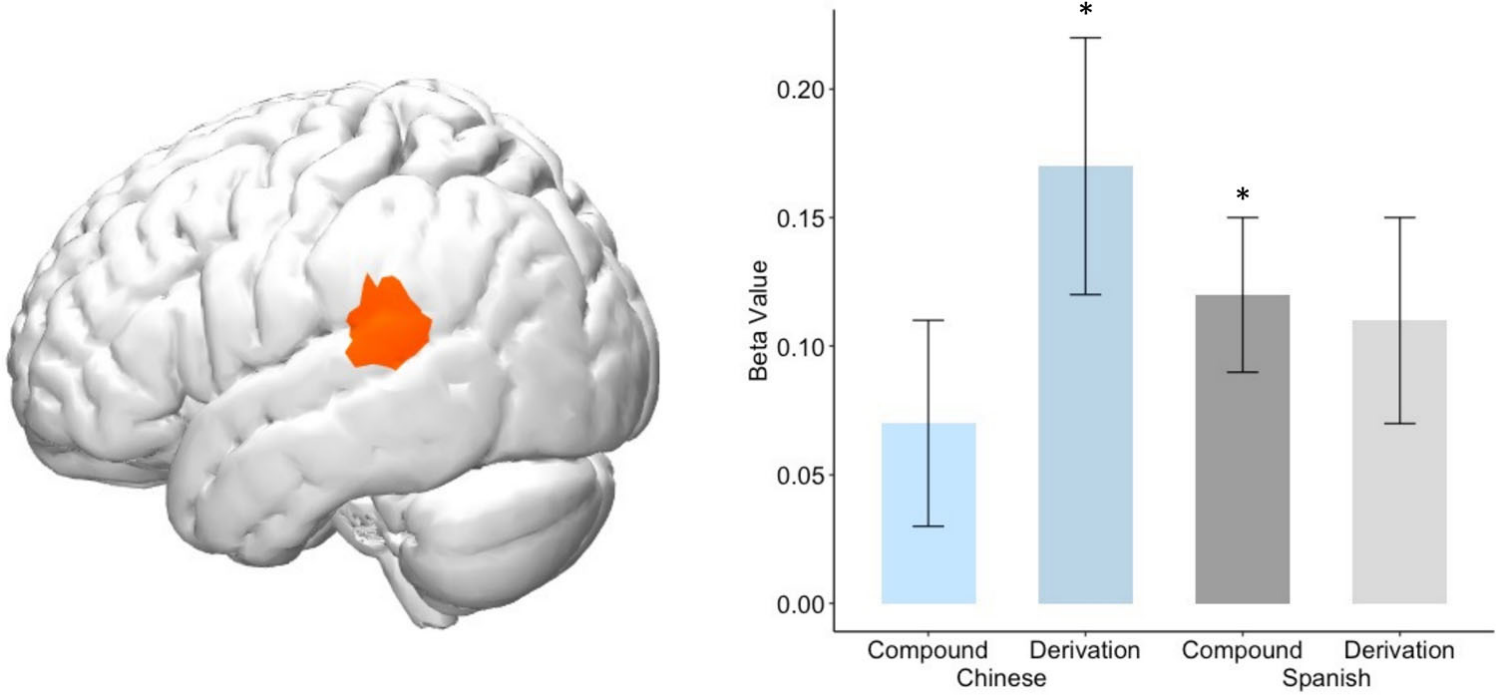
Brain‐behavior associations (M(SE) of beta values) revealed left STG activation in relation to heritage language proficiency by Free Roots/Compounds and Affixes/Derivations conditions (beta values compared to 0, *p ≤ 0.001)

In addition to examining the bilingual effects of experience with Chinese versus Spanish, we further asked about how individual differences in children's bilingual proficiency is related to neural functionality of English word processing across groups. To investigate the relation between bilinguals’ home language proficiency and their morphological processing in English, we conducted two separate GLMs for each bilingual group and modeled the interactions between home language vocabulary and morphological task condition, controlling for English vocabulary. These analyses were exploratory and thus took a *p* threshold at 0.001, uncorrected. Higher Chinese proficiency was associated with stronger left STG (Ch 9, Figure 5) activation during the affixes/derivations condition (*β* = 0.17, *SE* = 0.05, *t* = 3.54, *p* < 0.001, FDR‐corrected *q* = 0.021, Figure 5). Higher Spanish proficiency was associated with stronger left STG (Ch 9) activation during the root/compound condition (*β* = 0.12, *SE* = 0.04, *t* = 3.28, *p* = 0.001, FDR‐corrected *q* = 0.102, Figure 5). There were no significant associations between home language proficiency and brain activation during the root/compound condition in Chinese (*β* = 0.07, *SE* = 0.04, *t* = 1.63, *p* = 0.104, FDR‐corrected *q* = 0.481, Figure 5) or derivation condition in Spanish (*β* = 0.11, *SE* = 0.04, *t* = 2.96, *p* = 0.003, FDR‐corrected *q* = 0.301, Figure 5).

## 4 DISCUSSION

How do early bilingual experiences influence children's neural architecture for word processing? To answer this question, we examined lexical morphology processes in the English language in bilingual heritage speakers of structurally distinct languages—Spanish and Chinese—in relation to each other as well as English monolinguals. The focus on lexical morphology was motivated by the cross‐linguistic differences in morphological structures across the three languages that may help reveal the mechanisms guiding children's recognition of complex polysyllabic lexical items (Chung et al., 2019). Group comparisons revealed both *language‐specific* and *shared* bilingual effects. Regarding the former, the bilingual groups differed in their neural response to English words compared to each other and English monolinguals, and these differences can be attributed to specific morphological structures of their home languages. Regarding the latter, both bilingual groups showed a significant association between their home language proficiency and left STG brain activity when processing the English morphological structures that were most *distinct* from their home language. The findings inform theoretical perspectives on language and brain development by illuminating experience‐based plasticity in linguistically diverse learners.

### 4.1 Behavioral performance across linguistically diverse speakers

Developmental theories of bilingual transfer often cast lexical morphology development within the literacy frameworks (Chung et al., 2019). This is motivated by the growing evidence on the reciprocity in the growth of children's lexical morphology and reading skills in English. For instance, learning to read helps clarify the relation between morphemic units in a language, among other lexical skills (Carlisle, 1995). Therefore, in the present study, experimental groups were purposefully matched on English literacy skills, including single word reading and reading comprehension. The two bilingual groups were also matched in their English vocabulary which we generally expected to be lower than that of the monolinguals given the split lexical experience of the bilingual learners (Poulin‐Dubois et al., 2013). The resultant groups thus turned out to be similar across other literacy measures, including phonological and morphological awareness tasks.

In keeping with their equivalent performance on standardized behavioral measures, there were no significant group differences in the fNIRS morphological processing task accuracy. Across all three groups, the affixes/derivations condition was the most challenging; this is likely because this condition requires matching on more abstract, bound morphemes (e.g., *‐est, ‐ment*) than the free roots/compounds condition. Furthermore, there were no significant differences across groups in their accuracy on Germanic‐origin versus Latinate‐origin affixes. Both the Spanish‐English and Chinese‐English bilingual groups achieved higher accuracy on Germanic items than Latinate items, perhaps related to their slightly lower English vocabulary as compared to the monolinguals, and the fact that Latinate vocabulary items are typically acquired later (Hernandez et al., 2021). Even a close look at the Latinate derivations, the point of closest contact between English and Spanish, revealed no behavioral advantage of cross‐linguistic transfer for the Spanish‐English bilinguals. Nevertheless, we observed neurocognitive differences across bilingual groups and conditions.

### 4.2 Neurocognitive differences in morphology across linguistically diverse speakers

Polymorphemic words challenge listeners to consider the multifaceted elements of word sound, meaning, and structure (Gwilliams, 2020). Indeed, when processing both derived and compound multimorphemic words, all children showed robust activation in the left IFG region known for its analytical role in considering multiple complex elements of language structure (Hagoort, 2005; 2019). Yet, specific group and condition differences emerged, revealing a principled effect of language background on how proficient English speakers process different types of English words.

#### 4.2.1 English monolinguals

Monolingual children exhibited a robust engagement of the language network during the affixes/derivations condition relative to the free roots/compounds condition. In particular, the left IFG activation was present during the free roots/compounds condition, and it was more extensive and coupled with additional engagement of temporal, parietal, and occipital regions during the affixes/derivations condition. This observation that the affixes/derivations condition elicited greater neural engagement is commensurate with the developmental and adult neuroimaging work, as well as task performance evidence (Gwilliams, 2020; Leminen et al., 2019). This aligned with the behavioral indicators that all groups had better accuracy during the free roots/compounds relative to the affixes/derivations condition. Moreover, English compounding competence develops ahead of derivations (Marks, Eggleston, et al., 2021). Finally, whereas morphological awareness undergoes substantial finetuning during the school years, the present findings suggest that mechanisms supporting this development include an adult‐like engagement of the left‐lateralized language network (left IFG, STG, MTG), along with parietal and occipitotemporal regions (Gwilliams, 2020; Leminen et al., 2019).

#### 4.2.2 Chinese bilinguals

There were some notable similarities and differences in how Chinese bilinguals processed English morphology relative to monolinguals and Spanish‐English bilinguals. Similar to English monolinguals, left IFG activation in the Chinese bilinguals was present during the free roots/compounds condition and was more extensive and coupled with additional engagement of temporal, and parietal regions during the affixes/derivations condition. Nevertheless, a group comparison revealed during the affixes/derivations condition, Chinese bilinguals exhibited stronger left IFG/MFG activation and weaker parietal/angular gyrus (AG) activation than the monolinguals as well as Spanish‐English bilinguals. In comparison to Spanish bilinguals, Chinese bilinguals also exhibited stronger left STG and right frontal activation.

The greater engagement of bilateral IFG and left STG regions during the affixes/derivations condition is aligned with our prediction that Chinese bilinguals would demonstrate less automaticity when processing derivational structures as a result of the language‐specific bilingual transfer. In particular, affixes and derivations are not characteristic of the Chinese language. Therefore, across bilinguals’ combined dual‐language competencies, it is a lower‐frequency feature than in either monolinguals or Spanish bilinguals (Ramírez et al., 2011). As a lower‐frequency feature, affixes and derivations likely require additional analytical (left IFG) and word segmentation (left STG) resources on the part of the Chinese bilingual group. Notably, the Chinese bilinguals performed with equivalent accuracy in both the behavioral and the neuroimaging morphology tasks compared to the other groups. In sum, the findings suggest principled cross‐linguistic effects of Chinese bilingualism on children's neural organization, effects that potentially modify but do not impede bilingual word processing in English.

#### 4.2.3 Spanish bilinguals

A somewhat different pattern of results emerged in Spanish bilinguals despite their equivalent performance in the behavioral and neuroimaging morphology tasks as compared to the monolinguals and the Chinese bilinguals. Compared to the other groups, Spanish bilinguals had the fewest channels with significant differences between conditions, suggesting more similar neural processes. Intriguingly, their left IFG activation was stronger for the root/compound relative to the derived condition, which stands in contrast to the monolinguals and Chinese bilinguals. Furthermore, in contrast to the English monolinguals, during the affixes/derivations condition, Spanish bilinguals showed reduced right frontal and parietal activation relative to the monolinguals.

Spanish and English have more in common with each other morphologically than Chinese and English, and so the findings are more complex and difficult to interpret as more interactions can be expected in terms of both cross‐language facilitation and interference (Costa & Caramazza, 1999, 2000; Costa et al., 2005). In terms of facilitation, bilingual experiences with Spanish are often thought to facilitate derivational morphology processing in English due to the structural and lexical similarities of the affixation principles and the affixes themselves, which also helps obviate Latinate borrowings in English (Chung et al., 2019; Kuo et al., 2017; Ramírez et al., 2010, 2011). As a structurally similar high‐frequency feature across bilinguals’ two languages, derivational morphology may thus become more automated in the Spanish bilinguals, incurring less right hemisphere support relative to the English monolinguals as well as less bilateral frontal and left temporal activation relative to Chinese bilinguals.

Interference effects are also possible as there are several notable differences between Spanish and English compounding (Llorente, 2013; Ramírez et al., 2011). English compound morphology is relatively transparent, involving two root morphemes which often maintain their base form, as in *snow‐man*. In contrast, Spanish compounding is more analytically complex, often involving morpho‐phonological/syntactic modifications. For instance, *abrelatas* consists of *abrir* (to open) and *latas* (can), with the verb positioned before the noun. This structure is the opposite of English, in which compounds are generally right‐headed (e.g., N‐V as in *can‐open(er)*). Although compounding in both Chinese and Spanish is more complex than in English, Spanish‐English bilinguals likely build stronger interconnections between their two lexicons, yielding more points for cross‐linguistic facilitation and conflict (Chung et al., 2019). The greater frontotemporal activation for English compounding in Spanish bilinguals may therefore stem from their experience with the more analytically complex and structurally conflicting nature of Spanish compounding (Llorente, 2013; Ramírez et al., 2011).

### 4.3 Home language proficiency effects in bilingual's English

To examine the role of home proficiency on children's English, we tested the relation between bilinguals’ brain activity and home language proficiency, controlling for English proficiency (both measured with vocabulary). As expected, both groups demonstrated meaningful brain‐behavioral correlations with heritage language proficiency. Remarkably, the results of this whole‐brain analysis converged onto one region of the brain, left STG, revealing what might be a *shared* effect. For both bilingual groups, higher home language proficiency was associated with greater left STG activation in the condition that was *more dissimilar* to, or *less frequent* in their home language. Specifically, left STG activation was associated with *affixes/derivations condition* in Chinese bilinguals and *free roots/compounds condition* in Spanish bilinguals. Figure 5 suggests similar association strength (*beta* values) in STG for both conditions in Spanish, and this might be due to the fact that Spanish and English lexicons have much in common and thus co‐activation in bilinguals with more balanced proficiency affects all types of lexical constructions. However, it is notable that significance is only reached for the more dissimilar (i.e., root/compound) condition and the affixes/derivations condition was far from significant especially after multiple comparison correction (FDR‐corrected *q* = 0.301).

Left STG plays an essential role in word segmentation or analyzing words’ phonological and morphological constituents (Arredondo et al., 2015; Friederici & Gierhan, 2013; Friederici et al., 2003; Leminen et al., 2019; Leonard & Chang, 2014; Liebenthal et al., 2005; J. Wang et al., 2021). For instance, Arredondo et al. (2015) showed that children with stronger morphological awareness skills exhibited stronger left STG activation during a morphology task. Intriguingly, bilingual research also finds a pattern of greater STG activations in early‐exposed proficient bilinguals, as compared to later‐exposed or lower‐proficiency bilinguals (see the meta‐analysis by Cargnelutti et al., 2019), and this effect can be attributed to consistent dual‐language co‐activation demands from an early age (Chee et al., 2003; Kovelman et al., 2009). Advancing upon these generalized observations, we found an association between left STG activation and dual‐language proficiency in bilinguals with different language pairings. In sum, early bilingual experiences may influence brain development for language function by finetuning neural mechanisms of word recognition and segmentation.

These neuroimaging findings are also consistent with behavioral findings for this group in a separate inquiry (Marks, Eggleston, et al., 2021). This separate inquiry focused on the association between bilingual children's word reading and morphological awareness in English. The findings revealed that for both Spanish‐English and Chinese‐English bilingual children, children's awareness of English morphological features that were *more dissimilar* to, and *less frequent* in their home language predicted differences in their word reading proficiency. Notably, Marks, Labotka, et al. (2021) used a different task of morphological awareness that also tapped into derived versus compound morphological awareness. The converging neuro‐behavioral evidence reveals the underlying nature of dual language representations that consider both similarities and the differences of the two lexicons and how those are processed by the developing mind and brain.

### 4.4 Theoretical contributions: Language, bilingualism & the developing brain

Theories of bilingualism typically conceptualize bilingual processes as consisting of one set of shared language systems within which the two languages interact (Dijkstra et al., 2019). Within the bilingual system, connections are established through the simultaneous activations of related words (Kroll et al., 2010). As a result, starting from a young age, bilinguals often find themselves considering both within‐ and between‐language competitors when selecting a word (Arredondo et al., 2019; Shook & Marian, 2019). Our new bilingual findings demonstrate that these bilingual connections extend to sub‐lexical components, namely lexical morphology. In particular, we find that shared elements across a bilingual's two languages appear to gain automaticity, whereas those that are distinct appear to elicit a more effortful or otherwise attentive neural response. This was particularly evident in Spanish‐English bilinguals whose lexicons share many elements, potentially yielding reduced frontal activation for derivation (similar) and more temporal activation for compound (dissimilar) structures. We did not find automaticity/enhancement effects for compound processes in Chinese bilinguals, potentially because, while similar in principle, both the compounding processes and the lexical elements are quite distinct between English and Chinese. Yet, we did find stronger frontal activation for derivation structures that are disproportionately more characteristic of English than Chinese.

Importantly, brain‐behavior correlations provide a critical insight that helps bridge bilingual transfer and neuro‐cognitive perspectives of bilingualism across multiple language systems. Among our early‐exposed and proficient bilingual participants, those with stronger home language proficiency showed stronger STG activation for morphological structures that were most distinct between the two languages: derivation in Chinese and compounding in Spanish bilinguals. In accordance with transfer or cross‐linguistic adaptation perspectives, co‐activation of two languages results in careful modulation of language systems’ sensitivity towards both similar as well as dissimilar/unique features of each language (e.g., word order: Satterfield & Rusty, 2004). For instance, in mixed‐language utterances, bilingual children and adults often avoid mixing elements that may violate the structure of one of the two languages (Petitto & Kovelman, 2003; Zwanziger et al., 2005). Furthermore, neuro‐cognitive theories of bilingualism suggest that individuals with a longer history of bilingualism and stronger dual‐language proficiency should develop greater automaticity in attentional processes involved in dual language switching (UBET; DeLuca, 2020). The present findings revealed *shared* bilingual effects that early and proficient bilingualism finetunes core lexical segmentation processes, leading to greater automaticity of shared and sensitivity to unique linguistic elements. The imaging investigation allowed for findings of neuro‐cognitive commonalities among children with different bilingual experiences, yielding unique contributions to research on the impacts of early bilingualism.

### 4.5 Limitations

The present study has several caveats, including multiple cross‐cultural and socio‐linguistic differences between the experimental groups that include and extend beyond the language measurements in this study. For instance, monolinguals outperformed bilinguals in English vocabulary. Nevertheless, the observed neural differences between bilinguals and monolinguals are similar to those observed between the two bilingual groups, which supports our interpretation of cross‐linguistic transfer despite some proficiency differences. Another possible issue with the measurement is that, although we carefully chose the words of the morphological task based on age‐of‐acquisition indices and pilot results, we did not test the familiarity of the words for each participant. Nonetheless, participants performed with moderate to high accuracy and demonstrated no group‐level differences. Second, the wide age range of the participants contributes to the variability of bilingual effects. Nevertheless, the groups are well‐matched in their age and grade, and our findings are generally in line with neuroimaging findings for word processing in children (Arredondo et al., 2015; Ip et al., 2017) and adults (Leminen et al., 2019). Third, fNIRS analysis modeled trials within a block altogether, therefore the results were not able to capture children's neural functions for correct versus incorrect trials. However, the block design allowed for more robust data, and this is especially helpful for neuroimaging research with children. Future studies may adopt a different task design and examine how children's neural responses vary with individual items. Finally, since our samples were early‐exposed simultaneous bilingual children, our findings, therefore, are limited in informing the research on the critical periods in later‐exposed bilinguals. Future studies could seek to build a connection between the literature.

## 5 CONCLUSION

The present work aimed to uncover the effects of bilingualism on children's emerging neural architecture for language processing. The findings suggest an interaction between children's dual language experiences and proficiency in shaping bilingual development. English word structures that were most dissimilar or unique across bilinguals’ two languages elicited the greatest neural differences between the groups. In essence, the findings suggest an *interaction* between the *common* effects of bilingual proficiency and the *language‐specific* transfer such that shared structures gain automaticity of processing and distinct structures gain neuro‐cognitive sensitivity during the word segmentation processes. The findings illuminate the efficiency and plasticity of neural processes that make it possible to achieve successful dual‐language acquisition.

## CONFLICT OF INTEREST DISCLOSURE

We have no known conflict of interest to disclose.

## Supporting information



Supporting Information

## Data Availability

Data can be found via the OSF data manuscript https://osf.io/ybnps/?view_only=d14244924b264824896374ba8c7bd3d3.

## References

[desc13251-bib-0001] Abutalebi, J. , & Green, D. W. (2016). Neuroimaging of language control in bilinguals: Neural adaptation and reserve. Bilingualism: Language and cognition, 19(4), 689–698. 10.1017/S1366728916000225

[desc13251-bib-0002] Arredondo, M. M. , Hu, X. S. , Satterfield, T. , & Kovelman, I. (2017). Bilingualism alters children's frontal lobe functioning for attentional control. Developmental Science, 20(3), e12377. 10.1111/desc.12377 PMC5376242 26743118

[desc13251-bib-0003] Arredondo, M. M. , Hu, X. S. , Satterfield, T. , Riobóo, A. T. , Gelman, S. A. , & Kovelman, I. (2019). Bilingual effects on lexical selection: A neurodevelopmental perspective. Brain and Language, 195, 104640. 10.1016/j.bandl.2019.104640 31252177 PMC6716384

[desc13251-bib-0004] Arredondo, M. M. , Ip, K. I. , Shih Ju Hsu, L. , Tardif, T. , & Kovelman, I. (2015). Brain bases of morphological processing in young children. Human Brain Mapping, 36(8), 2890–2900. 10.1002/hbm.22815 25930011 PMC5374976

[desc13251-bib-0005] Aylward, E. H. , Richards, T. L. , Berninger, V. W. , Nagy, W. E. , Field, K. M. , Grimme, A. C. , Richards, A. L. , Thomson, J. B. , & Cramer, S. C. (2003). Instructional treatment associated with changes in brain activation in children with dyslexia. Neurology, 61(2), 212–219. 10.1212/01.WNL.0000068363.05974.64 12874401

[desc13251-bib-0006] Barker, J. W. , Aarabi, A. , & Huppert, T. J. (2013). Autoregressive model based algorithm for correcting motion and serially correlated errors in fNIRS. Biomedical Optics Express, 4(8), 1366–1379. 10.1364/BOE.4.001366 24009999 PMC3756568

[desc13251-bib-0009] Binder, J. R. (2017). Current controversies on Wernicke's area and its role in language. Current Neurology and Neuroscience Reports, 17, 58. 10.1007/s11910-017-0764-8 28656532

[desc13251-bib-0011] Cargnelutti, E. , Tomasino, B. , & Fabbro, F. (2019). Language brain representation in bilinguals with different age of appropriation and proficiency of the second language: A meta‐analysis of functional imaging studies. Frontiers in Human Neuroscience, 13, 154. 10.3389/fnhum.2019.00154 31178707 PMC6537025

[desc13251-bib-0012] Carlisle, J. F. (1995). Morphological awareness and early reading achievement. In ( L. Feldman Ed.), Morphological aspects of language processing (pp. 189–209). Erlbaum.

[desc13251-bib-0013] Chee, M. W. , Soon, C. S. , & Lee, H. L. (2003). Common and segregated neuronal networks for different languages revealed using functional magnetic resonance adaptation. Journal of Cognitive Neuroscience, 15(1), 85–97. 10.1162/089892903321107846 12590845

[desc13251-bib-0014] Chung, S. C. , Chen, X. , & Geva, E. (2019). Deconstructing and reconstructing cross‐language transfer in bilingual reading development: An interactive framework. Journal of Neurolinguistics, 50, 149–161. 10.1016/j.jneuroling.2018.01.003

[desc13251-bib-0015] Costa, A. , & Caramazza, A. (1999). Is lexical selection in bilingual speech production language‐specific? Further evidence from Spanish–English and English–Spanish bilinguals. Bilingualism: Language and cognition, 2(3), 231–244. 10.1017/S1366728999000334

[desc13251-bib-0016] Costa, A. , & Caramazza, A. (2000). Lexical access in speech production: The bilingual case. Psicológica, 21(2), 403–437. https://www.redalyc.org/articulo.oa?id=16921211

[desc13251-bib-0017] Costa, A. , Santesteban, M. , & Caño, A. (2005). On the facilitatory effects of cognate words in bilingual speech production. Brain and Language, 94(1), 94–103. 10.1016/j.bandl.2004.12.002 15896387

[desc13251-bib-0018] Das, T. , Padakannaya, P. , Pugh, K. R. , & Singh, N. C. (2011). Neuroimaging reveals dual routes to reading in simultaneous proficient readers of two orthographies. Neuroimage, 54(2), 1476–1487. 10.1016/j.neuroimage.2010.09.022 20854914 PMC3654519

[desc13251-bib-0019] Davies, M. (2020). The Corpus of Contemporary American English (COCA). Available online at https://www.english‐corpora.org/coca/

[desc13251-bib-0020] DeLuca, V. , Segaert, K. , Mazaheri, A. , & Krott, A. (2020). Understanding bilingual brain function and structure changes? U Bet! A Unified Bilingual Experience Trajectory model. Journal of Neurolinguistics, 56, 100930. 10.1016/j.jneuroling.2020.100930

[desc13251-bib-0021] Devlin, J. T. , Jamison, H. L. , Matthews, P. M. , & Gonnerman, L. M. (2004). Morphology and the internal structure of words. Proceedings of the National Academy of Sciences, 101(41), 14984–14988. 10.1073/pnas.0403766101 PMC522020 15358857

[desc13251-bib-0022] Dijkstra, T. , Wahl, A. , Buytenhuijs, F. , Van Halem, N. , Al‐Jibouri, Z. , De Korte, M. , & Rekké, S. (2019). Multilink: A computational model for bilingual word recognition and word translation. Bilingualism: Language and Cognition, 22(4), 657–679. 10.1017/S1366728918000287

[desc13251-bib-0023] Dunn, D. M. (2019). Peabody picture vocabulary test 5. NCS Pearson.

[desc13251-bib-0024] Dunn, L. , Padilla, E. , Lugo, D. , & Dunn, L. (1986). TVIP: Test vocabolario imágenes peabody. American Guidance Service.

[desc13251-bib-0025] Enge, A. , Friederici, A. D. , & Skeide, M. A. (2020). A meta‐analysis of fMRI studies of language comprehension in children. Neuroimage, 215, 116858. 10.1016/j.neuroimage.2020.116858 32304886

[desc13251-bib-0026] Ettinger, A. , Linzen, T. , & Marantz, A. (2014). The role of morphology in phoneme prediction: Evidence from MEG. Brain and Language, 129, 14–23. 10.1016/j.bandl.2013.11.004 24486600

[desc13251-bib-0027] Fiorentino, R. , & Poeppel, D. (2007). Compound words and structure in the lexicon. Language and Cognitive Processes, 22(7), 953–1000. 10.1080/01690960701190215

[desc13251-bib-0028] Friederici, A. D. , & Gierhan, S. M. (2013). The language network. Current Opinion in Neurobiology, 23(2), 250–254. 10.1016/j.conb.2012.10.002 23146876

[desc13251-bib-0029] Friederici, A. D. , Rüschemeyer, S. A. , Hahne, A. , & Fiebach, C. J. (2003). The role of left inferior frontal and superior temporal cortex in sentence comprehension: Localizing syntactic and semantic processes. Cerebral Cortex, 13(2), 170–177. 10.1093/cercor/13.2.170 12507948

[desc13251-bib-0030] Friston, K. J. , Ashburner, J. , Kiebel, S. J. , Nichols, T. , & Penny, W. (2007). Statistical parametric mapping: The analysis of functional brain images. Academic Press.

[desc13251-bib-0031] Gagnon, L. , Yücel, M. A. , Dehaes, M. , Cooper, R. J. , Perdue, K. L. , Selb, J. , Huppert, T. J. , Hoge, R. D. , & Boas, D. A. (2012). Quantification of the cortical contribution to the NIRS signal over the motor cortex using concurrent NIRS‐fMRI measurements. Neuroimage, 59(4), 3933–3940. 10.1016/j.neuroimage.2011.10.054 22036999 PMC3279595

[desc13251-bib-0032] Goh, W. D. , Yap, M. J. , & Chee, Q. W. (2020). The Auditory English Lexicon Project: A multi‐talker, multi‐region psycholinguistic database of 10,170 spoken words and nonwords. Behavior Research Methods, 52(5), 2202–2231. 10.3758/s13428-020-01352-0 32291734

[desc13251-bib-0033] Gwilliams, L. (2020). How the brain composes morphemes into meaning. Philosophical Transactions of the Royal Society B: Biological Sciences, 375(1791). Article 20190311. 10.1098/rstb.2019.0311 PMC6939360 31840591

[desc13251-bib-0034] Hagoort, P. (2005). On Broca, brain, and binding: A new framework. Trends in Cognitive Sciences, 9(9), 416–423. 10.1016/j.tics.2005.07.004 16054419

[desc13251-bib-0035] Hagoort, P. (2019). The neurobiology of language beyond single‐word processing. Science, 366(6461), 55–58. 10.1126/science.aax0289 31604301

[desc13251-bib-0037] Hernandez, A. E. , Ronderos, J. , Bodet, J. P. , Claussenius‐Kalman, H. , Nguyen, M. V. , & Bunta, F. (2021). German in childhood and Latin in adolescence: On the bidialectal nature of lexical access in English. Humanities and Social Sciences Communications, 8(1), 1–12. 10.1057/s41599-021-00836-4

[desc13251-bib-0088] Hickok, G. , & Poeppel, D. (2007). The cortical organization of speech processing. Nature Reviews Neuroscience, 8(5), 393–402. 10.1038/nrn2113 17431404

[desc13251-bib-0038] Hoshi, Y. (2007). Functional near‐infrared spectroscopy: Current status and future prospects. Journal of Biomedical Optics, 12(6), 062106. 10.1117/1.2804911 18163809

[desc13251-bib-0039] Hu, X. S. , Wagley, N. , Rioboo, A. T. , DaSilva, A. F. , & Kovelman, I. (2020). Photogrammetry‐based stereoscopic optode registration method for functional near‐infrared spectroscopy. Journal of Biomedical Optics, 25(9), 095001. 10.1117/1.JBO.25.9.095001 32880124 PMC7463164

[desc13251-bib-0090] Huppert, T. J. , Karim, H. , Lin, C. C. , Alqahtani, B. A. , Greenspan, S. L. , & Sparto, P. J. (2017). Functional imaging of cognition in an old‐old population: A case for portable functional near‐infrared spectroscopy. PloS One, 12(10), e0184918. 10.1371/journal.pone.0184918 29023452 PMC5638236

[desc13251-bib-0040] Ip, K. I. , Hsu, L. S. J. , Arredondo, M. M. , Tardif, T. , & Kovelman, I. (2017). Brain bases of morphological processing in Chinese‐English bilingual children. Developmental Science, 20(5), e12449. 10.1111/desc.12449 PMC5309206 27523024

[desc13251-bib-0042] Jasinska, K. K. , Berens, M. S. , Kovelman, I. , & Petitto, L. A. (2017). Bilingualism yields language‐specific plasticity in left hemisphere's circuitry for learning to read in young children. Neuropsychologia, 98, 34–45. 10.1016/j.neuropsychologia.2016.11.018 27894901

[desc13251-bib-0043] Jasinska, K. K. , & Petitto, L. A. (2013). How age of bilingual exposure can change the neural systems for language in the developing brain: A functional near infrared spectroscopy investigation of syntactic processing in monolingual and bilingual children. Developmental Cognitive Neuroscience, 6, 87–101. 10.1016/j.dcn.2013.06.005 23974273 PMC6987800

[desc13251-bib-0044] Jasińska, K. K. , & Petitto, L. A. (2014). Development of neural systems for reading in the monolingual and bilingual brain: New insights from functional near infrared spectroscopy neuroimaging. Developmental Neuropsychology, 39(6), 421–439. 10.1080/87565641.2014.939180 25144256

[desc13251-bib-0045] Kasparian, K. , Vespignani, F. , & Steinhauer, K. (2017). First language attrition induces changes in online morphosyntactic processing and re‐analysis: An ERP study of number agreement in complex Italian sentences. Cognitive Science, 41(7), 1760–1803. 10.1111/cogs.12450 27868225 PMC5638100

[desc13251-bib-0046] Ke, S. , Miller, R. T. , Zhang, D. , & Koda, K. (2021). Crosslinguistic sharing of morphological awareness in biliteracy development: A systematic review and meta‐analysis of correlation coefficients. Language Learning, 71(1), 8–54. 10.1111/lang.12429

[desc13251-bib-0048] Kirby, J. R. , & Bowers, P. N. (2017). Morphological instruction and literacy: Binding phonological, orthographic, and semantic features of words. In ( K. Cain , D. L. Compton , & R. K. Parrila Eds.), Studies in written language and literacy (Vol., 15, pp. 437–462). John Benjamins Publishing Company. 10.1075/swll.15.24kir

[desc13251-bib-0049] Kovelman, I. , Baker, S. A. , & Petitto, L. A. (2008). Age of first bilingual language exposure as a new window into bilingual reading development. Bilingualism: Language and Cognition, 11(2), 203–223. 10.1017/S1366728908003386 19823598 PMC2759761

[desc13251-bib-0050] Kovelman, I. , Shalinsky, M. H. , White, K. S. , Schmitt, S. N. , Berens, M. S. , Paymer, N. , & Petitto, L. A. (2009). Dual language use in sign‐speech bimodal bilinguals: FNIRS brain‐imaging evidence. Brain and Language, 109(2‐3), 112–123. 10.1016/j.bandl.2008.09.008 18976807 PMC2749876

[desc13251-bib-0051] Kroll, J. F. , Van Hell, J. G. , Tokowicz, N. , & Green, D. W. (2010). The revised hierarchical model: A critical review and assessment. Bilingualism: Language and Cognition, 13(3), 373–381. 10.1017/S136672891000009X 20676387 PMC2910435

[desc13251-bib-0052] Kuo, L. J. , Ramírez, G. , de Marin, S. , Kim, T. J. , & Unal‐Gezer, M. (2017). Bilingualism and morphological awareness: A study with children from general education and Spanish‐English dual language programs. Educational Psychology, 37(2), 94–111. 10.1080/01443410.2015.1049586

[desc13251-bib-0053] Leminen, A. , Smolka, E. , Dunabeitia, J. A. , & Pliatsikas, C. (2019). Morphological processing in the brain: The good (inflection), the bad (derivation) and the ugly (compounding). Cortex; A Journal Devoted to the Study of the Nervous System and Behavior, 116, 4–44. 10.1016/j.cortex.2018.08.016 30268324

[desc13251-bib-0054] Leonard, M. K. , & Chang, E. F. (2014). Dynamic speech representations in the human temporal lobe. Trends in Cognitive Sciences, 18(9), 472–479. 10.1016/j.tics.2014.05.001 24906217 PMC4149812

[desc13251-bib-0055] Liebenthal, E. , Binder, J. R. , Spitzer, S. M. , Possing, E. T. , & Medler, D. A. (2005). Neural substrates of phonemic perception. Cerebral Cortex, 15(10), 1621–1631. 10.1093/cercor/bhi040 15703256

[desc13251-bib-0056] Liu, H. , & Cao, F. (2016). L1 and L2 processing in the bilingual brain: A meta‐analysis of neuroimaging studies. Brain and Language, 159, 60–73. 10.1016/j.bandl.2016.05.013 27295606

[desc13251-bib-0057] Liu, P. D. , & McBride‐Chang, C. (2010). What is morphological awareness? Tapping lexical compounding awareness in Chinese third graders. Journal of Educational Psychology, 102(1), 62–73. 10.1037/a0016933

[desc13251-bib-0089] Louleli, N. , Hämäläinen, J. A. , Nieminen, L. , Parviainen, T. , & Leppänen, P. H. T. (2020). Dynamics of morphological processing in pre‐school children with and without familial risk for dyslexia. Journal of Neurolinguistics, 56, 100931. 10.1016/j.jneuroling.2020.100931

[desc13251-bib-0058] Llorente, M. A. (2013). A comparative Aanalysis between English and Spanish native speakers’ production and comprehension of N‐N compounds. ES Review. Spanish Journal of English Studies, 34, 7–40. https://revistas.uva.es/index.php/esreview/article/view/2172

[desc13251-bib-0059] Lu, L. , & Liu, H. S. (1998). The peabody picture vocabulary test‐revised in Chinese. Psychological Publishing.

[desc13251-bib-0060] Luo, Y. C. , Chen, X. , & Geva, E. (2014). Concurrent and longitudinal cross‐linguistic transfer of phonological awareness and morphological awareness in Chinese‐English bilingual children. Written Language and Literacy, 17(1), 89–115. 10.1075/wll.17.1.05luo

[desc13251-bib-0061] Marks, R. A. , Eggleston, R. L. , Sun, X. , Yu, C.‐L. , Zhang, K. , Nickerson, N. , Hu, X.‐S. H. , & Kovelman, I. (2021). The neurocognitive basis of morphological processing in typical and impaired readers. Annals of Dyslexia, 10.1007/s11881-021-00239-9 PMC9663212 34255265

[desc13251-bib-0062] Marks, R. A. , Labotka, D. , Sun, X. , Nickerson, N. , Zhang, K. , Eggleston, R. L. , Yu, C. , Hoeft, F. U. Y. , & Kovelman, I. (2021). Morphological awareness contributes to early literacy in linguistically diverse readers. 10.31234/osf.io/xpycj

[desc13251-bib-0063] Pasquarella, A. , Chen, X. , Lam, K. , Luo, Y. C. , & Ramírez, G. (2011). Cross‐language transfer of morphological awareness in Chinese–English bilinguals. Journal of Research in Reading, 34(1), 23–42. 10.1111/j.1467-9817.2010.01484.x

[desc13251-bib-0065] Petitto, L. A. , & Kovelman, I. (2003). The bilingual paradox: How signing‐speaking bilingual children help us to resolve it and teach us about the brain's mechanisms underlying all language acquisition. Learning Languages, 8(3), 5–18.

[desc13251-bib-0066] Poulin‐Dubois, D. , Bialystok, E. , Blaye, A. , Polonia, A. , & Yott, J. (2013). Lexical access and vocabulary development in very young bilinguals. International Journal of Bilingualism, 17(1), 57–70. 10.1177/1367006911431198 PMC3992973 24761135

[desc13251-bib-0067] Ramírez, G. , Chen, X. , Geva, E. , & Kiefer, H. (2010). Morphological awareness in Spanish‐speaking English language learners: Within and cross‐language effects on word reading. Reading and Writing, 23(3), 337–358. 10.1007/s11145-009-9203-9

[desc13251-bib-0068] Ramírez, G. , Chen, X. , Geva, E. , & Luo, Y. (2011). Morphological awareness and word reading in English language learners: Evidence from Spanish‐and Chinese‐speaking children. Applied Psycholinguistics, 32(3), 601–618. 10.1017/S0142716411000233

[desc13251-bib-0069] Rupawala, M. , Dehghani, H. , Lucas, S. J. , Tino, P. , & Cruse, D. (2018). Shining a light on awareness: A review of functional near‐infrared spectroscopy for prolonged disorders of consciousness. Frontiers in Neurology, 9(350),. 10.3389/fneur.2018.00350 PMC5972220 29872420

[desc13251-bib-0070] Santosa, H. , Zhai, X. , Fishburn, F. , & Huppert, T. (2018). The NIRS brain AnalyzIR toolbox. Algorithms, 11(5), 73. 10.3390/a11050073 PMC11218834 38957522

[desc13251-bib-0071] Satterfield, T. , & Rusty, B. (2004). Generation Gap: Explaining new and emerging word‐order phenomena in Mayan‐Spanish bilinguals. Proceedings of the First International Symposium on Bilingualism in Latin America . 285–300. http://www‐personal.umich.edu/~tsatter/ESSARP04.pdf

[desc13251-bib-0072] Schrank, F. A. , McGrew, K. S. , & Mather, N. (2014). Woodcock‐Johnson IV. Riverside.

[desc13251-bib-0073] Shook, A. , & Marian, V. (2019). Covert co‐activation of bilinguals’ non‐target language: Phonological competition from translations. Linguistic Approaches to Bilingualism, 9(2), 228–252. 10.1075/lab.17022.sho 31367262 PMC6668354

[desc13251-bib-0074] Strangman, G. , Culver, J. P. , Thompson, J. H. , & Boas, D. A. (2002). A quantitative comparison of simultaneous BOLD fMRI and NIRS recordings during functional brain activation. Neuroimage, 17(2), 719–731. 10.1006/nimg.2002.1227 12377147

[desc13251-bib-0075] Sun, X. , Zhang, K. , Marks, R. , Nickerson, N. , Eggleston, R. , Yu, C. L. , Chou, T. , Tardif, T. , & Kovelman, I. (2021). What's in a word? Cross‐linguistic influences on Spanish‐English and Chinese‐English bilingual children's word reading development. Child Development, 10.1111/cdev.13666 PMC8766884 34570366

[desc13251-bib-0076] Tong, X. , Chung, K. K. H. , & McBride, C. (2014). Two‐character Chinese compound word processing in Chinese children with and without dyslexia: ERP evidence. Developmental Neuropsychology, 39(4), 285–301. 10.1080/87565641.2014.907720 24854773

[desc13251-bib-0077] Tong, X. , Deacon, S. H. , & Cain, K. (2014). Morphological and syntactic awareness in poor comprehenders: Another piece of the puzzle. Journal of Learning Disabilities, 47(1), 22–33. 10.1177/0022219413509971 24306458

[desc13251-bib-0078] Tong, X. , Deacon, S. H. , Kirby, J. R. , Cain, K. , & Parilla, R. (2011). Morphological awareness: A key to understanding poor reading comprehension in English. Journal of Educational Psychology, 103(3), 523–534. 10.1037/a0023495

[desc13251-bib-0087] Tong, X. , Tong, X. , & McBride, C. (2017). Unpacking the relation between morphological awareness and Chinese word reading: Levels of morphological awareness and vocabulary. Contemporary Educational Psychology, 48, 167–178. 10.1016/j.cedpsych.2016.07.003

[desc13251-bib-0079] Vannest, J. , Newport, E. L. , Newman, A. J. , & Bavelier, D. (2011). Interplay between morphology and frequency in lexical access: The case of the base frequency effect. Brain Research, 1373, 144–159. 10.1016/j.brainres.2010.12.022 21167136 PMC3038557

[desc13251-bib-0080] Wagner, R. K. , Torgesen, J. K. , Rashotte, C. A. , & Pearson, N. A. (1999). Comprehensive test of phonological processing: CTOPP. Pro‐ed.

[desc13251-bib-0081] Wang, M. , Cheng, C. X. , & Chen, S. W. (2006). Contribution of morphological awareness to Chinese‐English biliteracy acquisition. Journal of Educational Psychology, 98(3), 542–553. 10.1037/0022-0663.98.3.542

[desc13251-bib-0082] Wang, J. , Yamasaki, B. L. , Weiss, Y. , & Booth, J. R. (2021). Both frontal and temporal cortex exhibit phonological and semantic specialization during spoken language processing in 7‐to 8‐year‐old children. Human Brain Mapping, 42(11):3534–3546. 10.1002/hbm.25450 33951259 PMC8249890

[desc13251-bib-0083] Werker, J. F. , & Hensch, T. K. (2015). Critical periods in speech perception: New directions. Annual Review of Psychology, 66(1), 173–196. 10.1146/annurev-psych-010814-015104 25251488

[desc13251-bib-0085] Zwanziger, E. E. , Allen, S. E. , & Genesee, F. (2005). Crosslinguistic influence in bilingual acquisition: Subject omission in learners of Inuktitut and English. Journal of Child Language, 32(4), 893–909. 10.1017/S0305000905007129 16429716

