# Supplementary material for: Brain bases of English morphological processing: A comparison between Chinese‐English, Spanish‐English bilingual, and English monolingual children
Source: Dev Sci. 2022 Mar 1;26(1):e13251. doi: 10.1111/desc.13251 (PMC9615011; doi:10.1111/desc.13251)
Supplement: Supplementary file 1 — Supporting Information [file DESC-26-0-s001.docx]

***Supplementary Materials to manuscript***

**Brain bases of English morphological processing: A comparison between**

**Chinese-English, Spanish-English bilingual, and English monolingual children**

**Figure S1**

Participants’ Bilateral Brain Activation during Lexical Compound and Derivational Affixes Conditions (task > control) and direct comparisons of the two conditions (task > rest contrasts compared; all FDR adjusted *q* < .05)


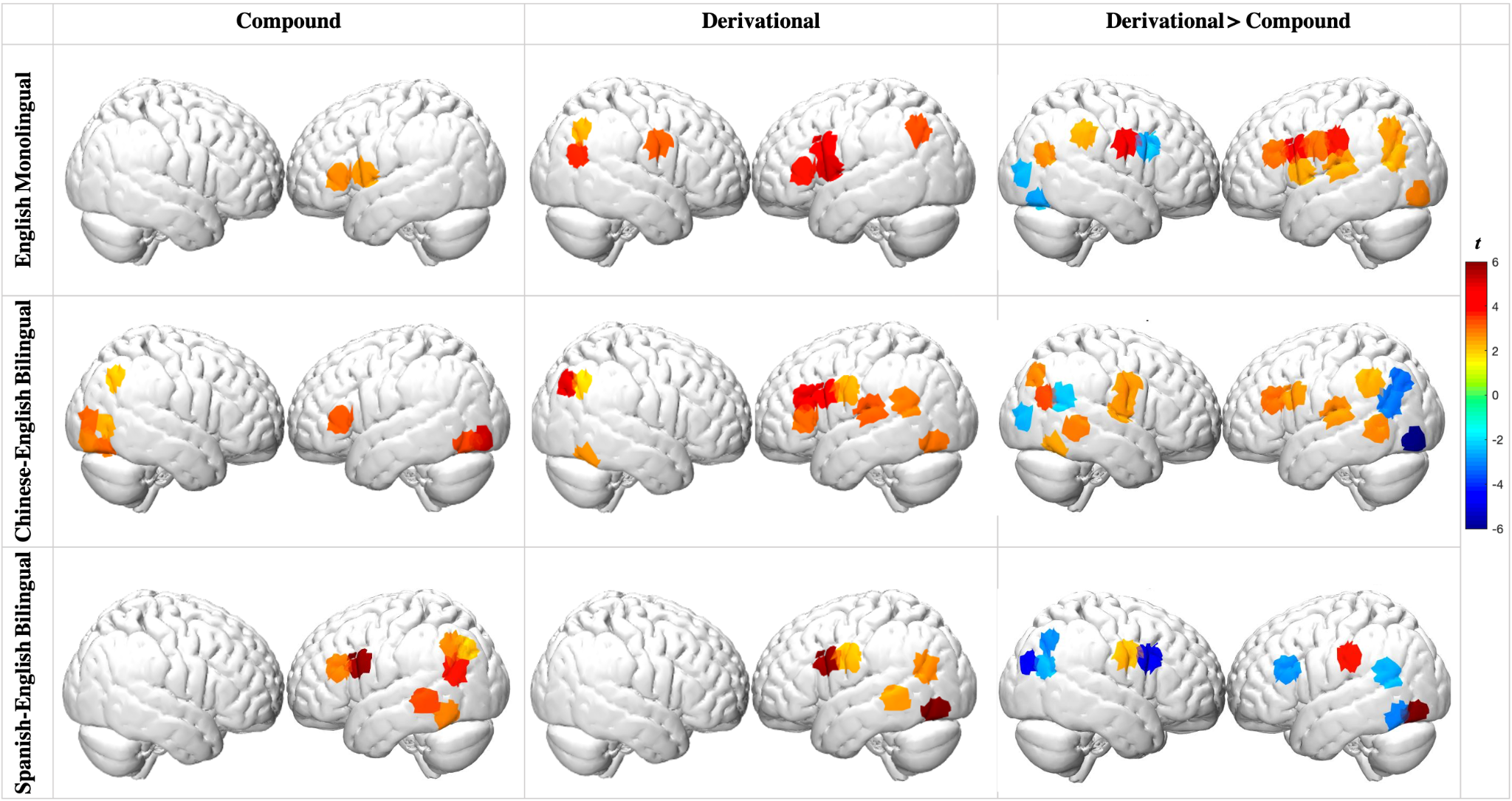


**Table S1**

*English Morphological Processing Task Stimuli (Correct answers are bolded)*

**
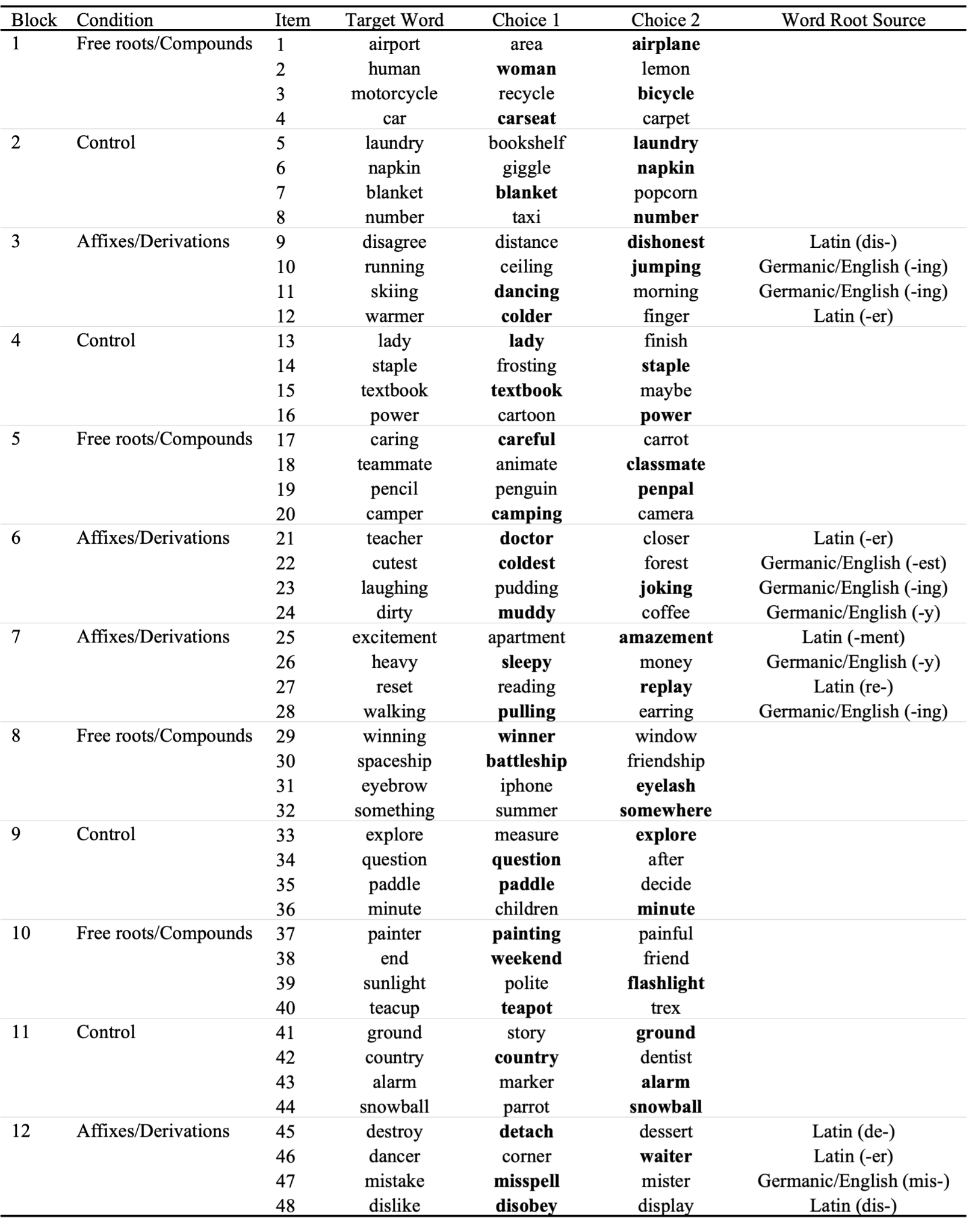
**

**Table S2**

*Brain Activations during the Lexical Compound Condition Relative to the Whole Word Control*
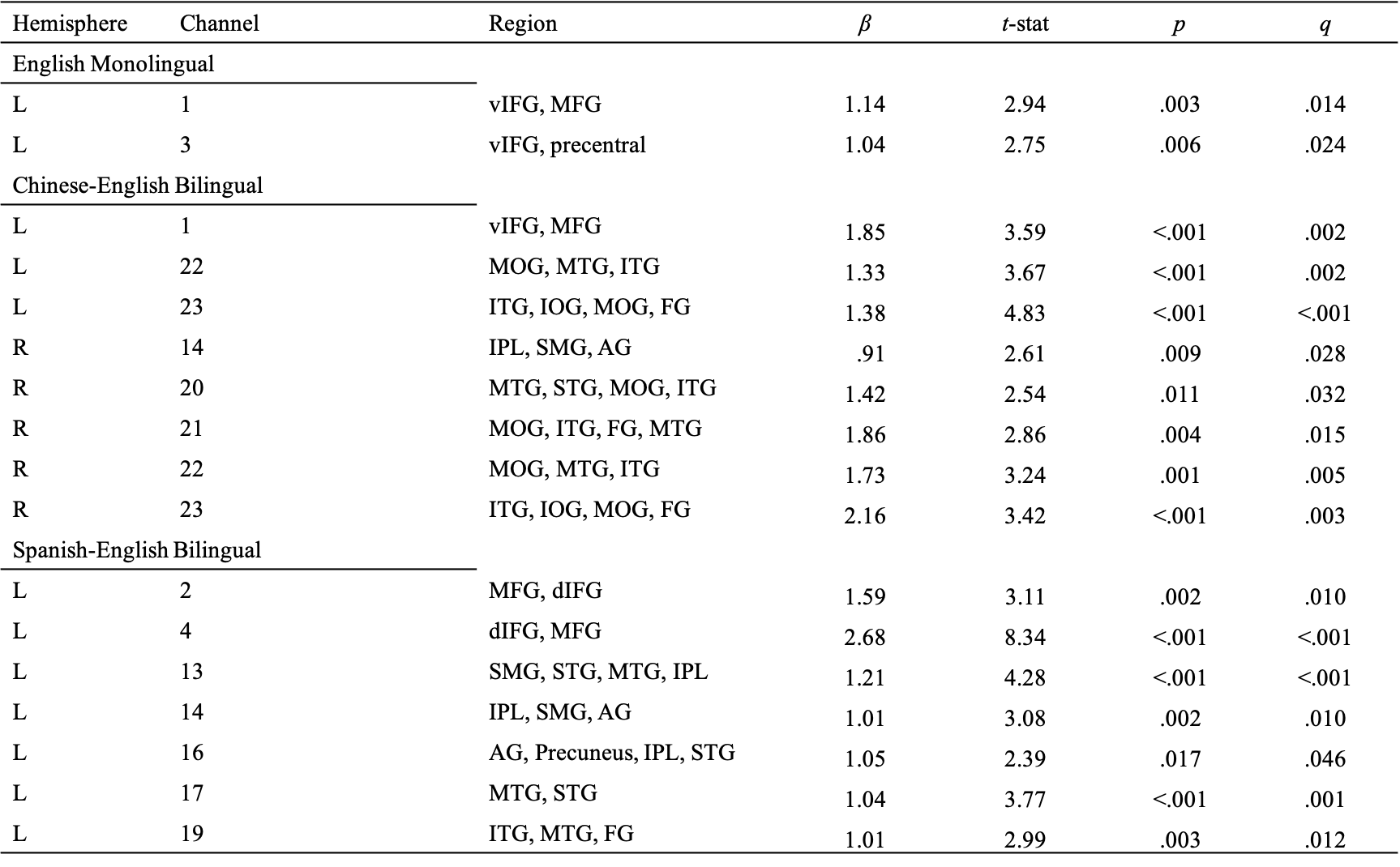


**Table S3**

*Brain Activations during the Derivational Affixes Condition Relative to the Whole Word Control*

***
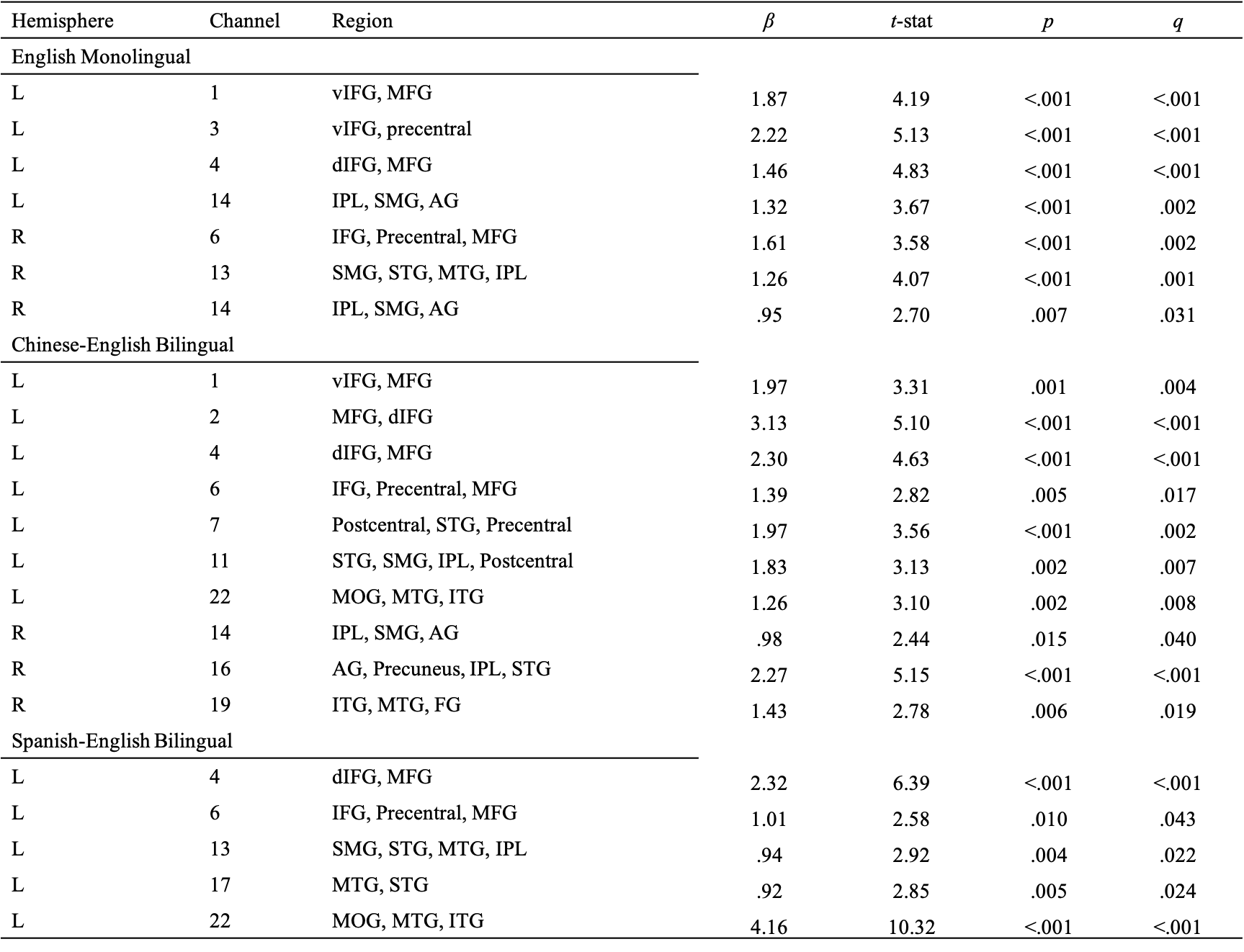
***

**Table S4**

*Brain Activations during the Derivational Affixes Condition Relative to the Lexical Compound Condition*

***
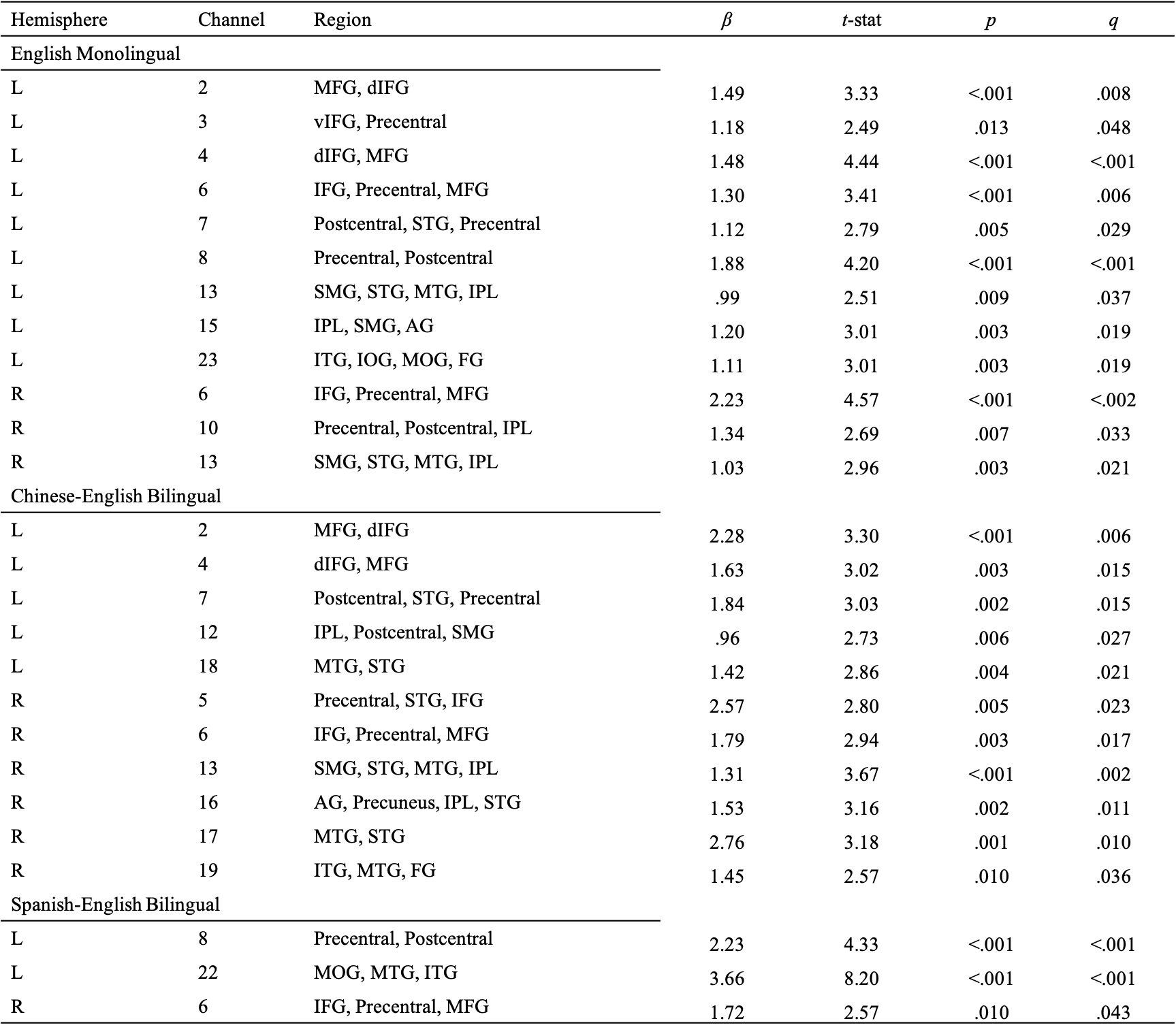
***

**Table S5**

*Brain Activations during the Lexical Compound Condition Relative to the Derivational Affixes Condition*

***
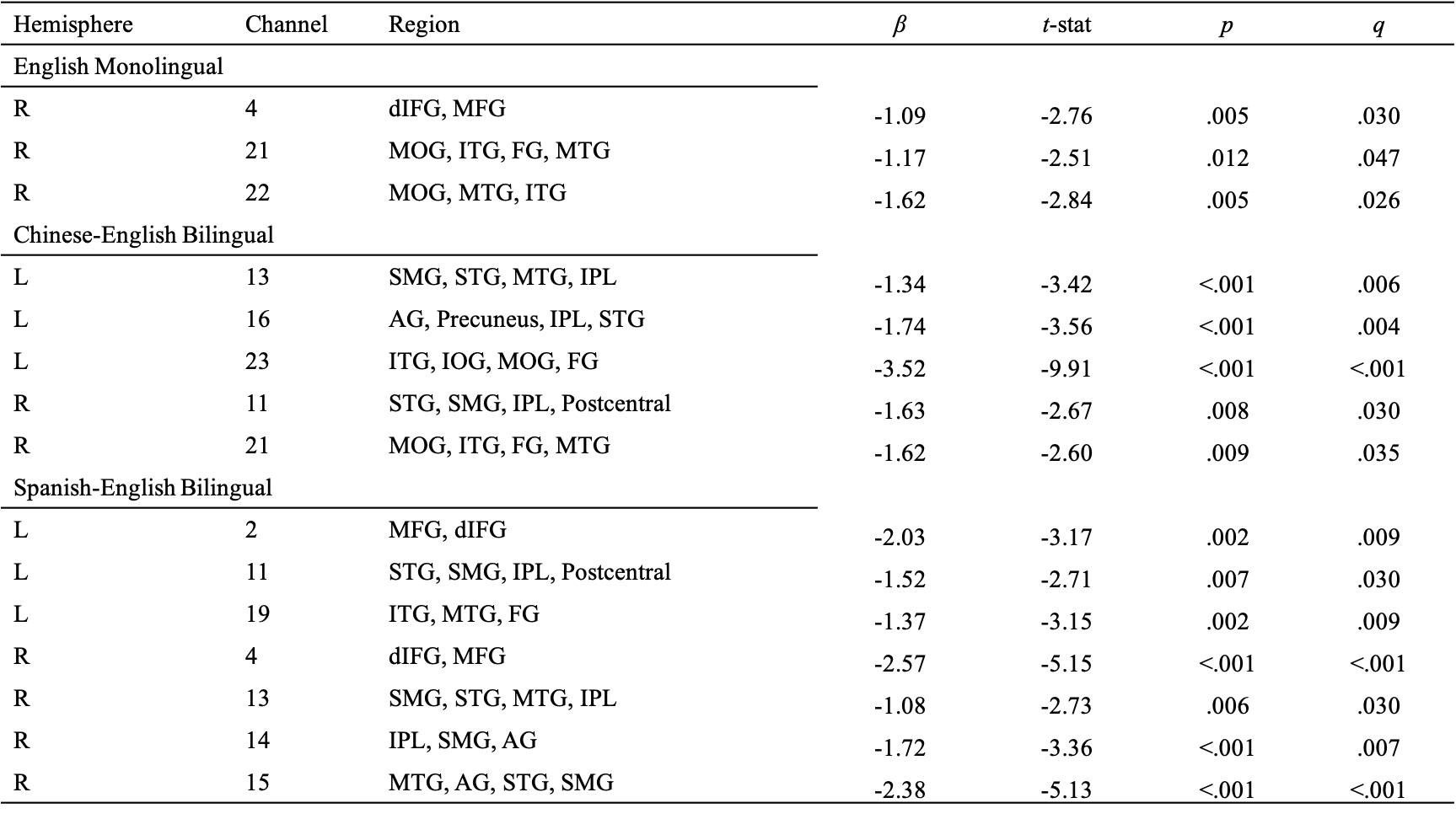
***

**Table S6**

*Brain Activations Across Language Group by Morphological Task Condition*

*
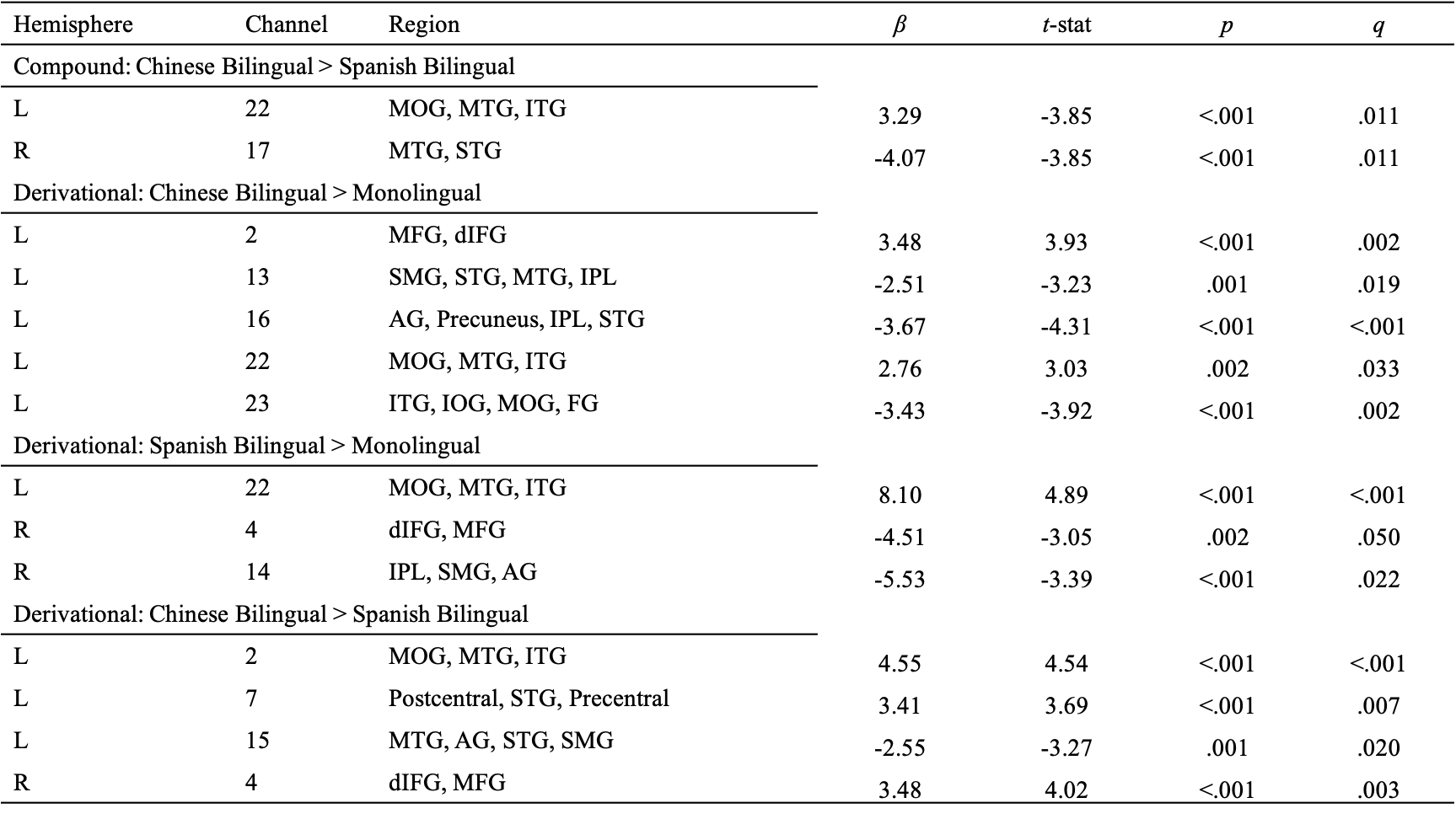
*
