# Supplementary material for: Brain bases of English morphological processing: A comparison between Chinese-English, Spanish-English bilingual, and English monolingual children
Source: Dev Sci. Author manuscript; Available in PMC 2024 Jan 1. (PMC9615011; doi:10.1111/desc.13251)
Supplement: Supplementary file 1 [file NIHMS1842574-supplement-Dev_Science_Supplement.pdf]

Supplementary Materials to manuscript
Brain bases of English morphological processing: A comparison between Chinese-English, Spanish-English bilingual, and English monolingual children

Figure S1

Participants' Bilateral Brain Activation during Lexical Compound and Derivational Affixes Conditions (task > control) and direct comparisons of the two conditions (task > rest contrasts compared; all FDR adjusted $q < .05$)

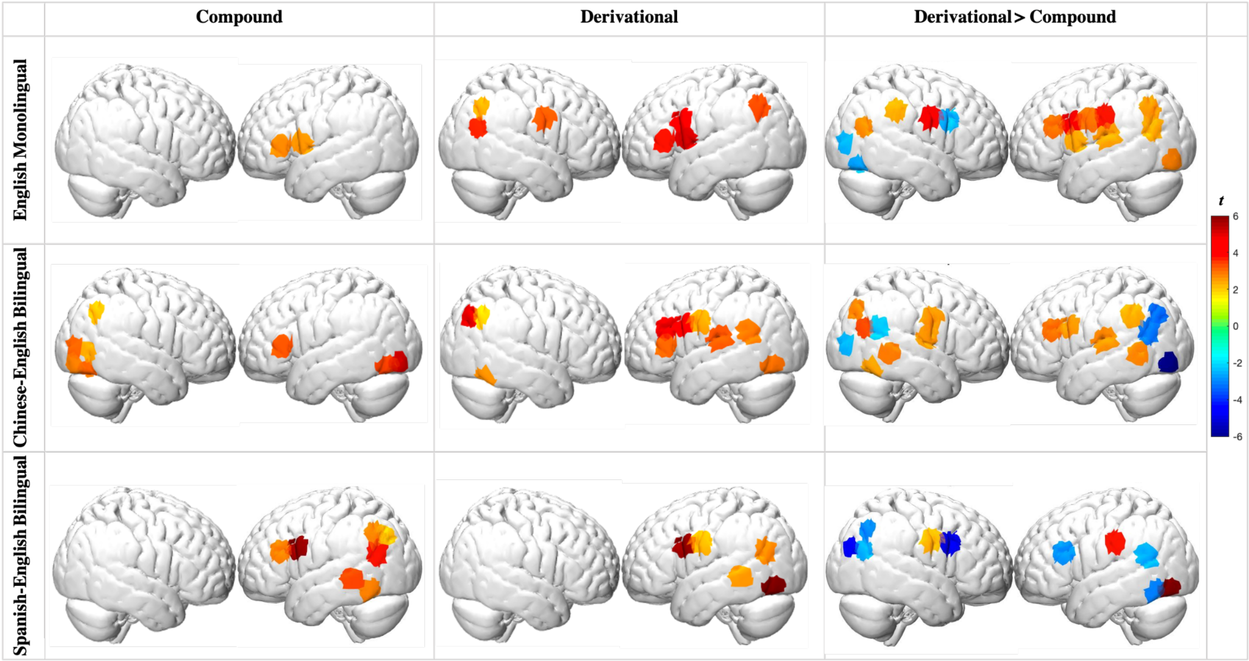

Table S1

English Morphological Processing Task Stimuli (Correct answers are bolded)

Block	Condition	Item	Target Word	Choice 1	Choice 2	Word Root Source
1	Free roots/Compounds	1	airport	area	**airplane**	
		2	human	**woman**	lemon	
		3	motorcycle	recycle	**bicycle**	
		4	car	**carseat**	carpet	
2	Control	5	laundry	bookshelf	**laundry**	
		6	napkin	giggle	**napkin**	
		7	blanket	**blanket**	popcorn	
		8	number	taxi	**number**	
3	Affixes/Derivations	9	disagree	distance	**dishonest**	Latin (dis-)
		10	running	ceiling	**jumping**	Germanic/English (-ing)
		11	skiing	**dancing**	morning	Germanic/English (-ing)
		12	warmer	**colder**	finger	Latin (-er)
4	Control	13	lady	**lady**	finish	
		14	staple	frosting	**staple**	
		15	textbook	**textbook**	maybe	
		16	power	cartoon	**power**	
5	Free roots/Compounds	17	caring	**careful**	carrot	
		18	teammate	animate	**classmate**	
		19	pencil	penguin	**penpal**	
		20	camper	**camping**	camera	
6	Affixes/Derivations	21	teacher	**doctor**	closer	Latin (-er)
		22	cutest	**coldest**	forest	Germanic/English (-est)
		23	laughing	pudding	**joking**	Germanic/English (-ing)
		24	dirty	**muddy**	coffee	Germanic/English (-y)
7	Affixes/Derivations	25	excitement	apartment	**amazement**	Latin (-ment)
		26	heavy	**sleepy**	money	Germanic/English (-y)
		27	reset	reading	**replay**	Latin (re-)
		28	walking	**pulling**	earring	Germanic/English (-ing)
8	Free roots/Compounds	29	winning	**winner**	window	
		30	spaceship	**battleship**	friendship	
		31	eyebrow	iphone	**eyelash**	
		32	something	summer	**somewhere**	
9	Control	33	explore	measure	**explore**	
		34	question	**question**	after	
		35	paddle	**paddle**	decide	
		36	minute	children	**minute**	
10	Free roots/Compounds	37	painter	**painting**	painful	
		38	end	**weekend**	friend	
		39	sunlight	polite	**flashlight**	
		40	teacup	**teapot**	trex	
11	Control	41	ground	story	**ground**	
		42	country	**country**	dentist	
		43	alarm	marker	**alarm**	
		44	snowball	parrot	**snowball**	
12	Affixes/Derivations	45	destroy	**detach**	dessert	Latin (de-)
		46	dancer	corner	**waiter**	Latin (-er)
		47	mistake	**misspell**	mister	Germanic/English (mis-)
		48	dislike	**disobey**	display	Latin (dis-)

Table S2

Brain Activations during the Lexical Compound Condition Relative to the Whole Word Control

Hemisphere	Channel	Region	*β*	*t*-stat	*p*	*q*
English Monolingual						
L	1	vIFG, MFG	1.14	2.94	.003	.014
L	3	vIFG, precentral	1.04	2.75	.006	.024
Chinese-English Bilingual						
L	1	vIFG, MFG	1.85	3.59	<.001	.002
L	22	MOG, MTG, ITG	1.33	3.67	<.001	.002
L	23	ITG, IOG, MOG, FG	1.38	4.83	<.001	<.001
R	14	IPL, SMG, AG	.91	2.61	.009	.028
R	20	MTG, STG, MOG, ITG	1.42	2.54	.011	.032
R	21	MOG, ITG, FG, MTG	1.86	2.86	.004	.015
R	22	MOG, MTG, ITG	1.73	3.24	.001	.005
R	23	ITG, IOG, MOG, FG	2.16	3.42	<.001	.003
Spanish-English Bilingual						
L	2	MFG, dIFG	1.59	3.11	.002	.010
L	4	dIFG, MFG	2.68	8.34	<.001	<.001
L	13	SMG, STG, MTG, IPL	1.21	4.28	<.001	<.001
L	14	IPL, SMG, AG	1.01	3.08	.002	.010
L	16	AG, Precuneus, IPL, STG	1.05	2.39	.017	.046
L	17	MTG, STG	1.04	3.77	<.001	.001
L	19	ITG, MTG, FG	1.01	2.99	.003	.012

Table S3

Brain Activations during the Derivational Affixes Condition Relative to the Whole Word Control

Hemisphere	Channel	Region	*β*	*t*-stat	*p*	*q*
English Monolingual						
L	1	vIFG, MFG	1.87	4.19	<.001	<.001
L	3	vIFG, precentral	2.22	5.13	<.001	<.001
L	4	dIFG, MFG	1.46	4.83	<.001	<.001
L	14	IPL, SMG, AG	1.32	3.67	<.001	.002
R	6	IFG, Precentral, MFG	1.61	3.58	<.001	.002
R	13	SMG, STG, MTG, IPL	1.26	4.07	<.001	.001
R	14	IPL, SMG, AG	.95	2.70	.007	.031
Chinese-English Bilingual						
L	1	vIFG, MFG	1.97	3.31	.001	.004
L	2	MFG, dIFG	3.13	5.10	<.001	<.001
L	4	dIFG, MFG	2.30	4.63	<.001	<.001
L	6	IFG, Precentral, MFG	1.39	2.82	.005	.017
L	7	Postcentral, STG, Precentral	1.97	3.56	<.001	.002
L	11	STG, SMG, IPL, Postcentral	1.83	3.13	.002	.007
L	22	MOG, MTG, ITG	1.26	3.10	.002	.008
R	14	IPL, SMG, AG	.98	2.44	.015	.040
R	16	AG, Precuneus, IPL, STG	2.27	5.15	<.001	<.001
R	19	ITG, MTG, FG	1.43	2.78	.006	.019
Spanish-English Bilingual						
L	4	dIFG, MFG	2.32	6.39	<.001	<.001
L	6	IFG, Precentral, MFG	1.01	2.58	.010	.043
L	13	SMG, STG, MTG, IPL	.94	2.92	.004	.022
L	17	MTG, STG	.92	2.85	.005	.024
L	22	MOG, MTG, ITG	4.16	10.32	<.001	<.001

Table S4

Brain Activations during the Derivational Affixes Condition Relative to the Lexical Compound Condition

Hemisphere	Channel	Region	*β*	*t*-stat	*p*	*q*
English Monolingual						
L	2	MFG, dIFG	1.49	3.33	<.001	.008
L	3	vIFG, Precentral	1.18	2.49	.013	.048
L	4	dIFG, MFG	1.48	4.44	<.001	<.001
L	6	IFG, Precentral, MFG	1.30	3.41	<.001	.006
L	7	Postcentral, STG, Precentral	1.12	2.79	.005	.029
L	8	Precentral, Postcentral	1.88	4.20	<.001	<.001
L	13	SMG, STG, MTG, IPL	.99	2.51	.009	.037
L	15	IPL, SMG, AG	1.20	3.01	.003	.019
L	23	ITG, IOG, MOG, FG	1.11	3.01	.003	.019
R	6	IFG, Precentral, MFG	2.23	4.57	<.001	<.002
R	10	Precentral, Postcentral, IPL	1.34	2.69	.007	.033
R	13	SMG, STG, MTG, IPL	1.03	2.96	.003	.021
Chinese-English Bilingual						
L	2	MFG, dIFG	2.28	3.30	<.001	.006
L	4	dIFG, MFG	1.63	3.02	.003	.015
L	7	Postcentral, STG, Precentral	1.84	3.03	.002	.015
L	12	IPL, Postcentral, SMG	.96	2.73	.006	.027
L	18	MTG, STG	1.42	2.86	.004	.021
R	5	Precentral, STG, IFG	2.57	2.80	.005	.023
R	6	IFG, Precentral, MFG	1.79	2.94	.003	.017
R	13	SMG, STG, MTG, IPL	1.31	3.67	<.001	.002
R	16	AG, Precuneus, IPL, STG	1.53	3.16	.002	.011
R	17	MTG, STG	2.76	3.18	.001	.010
R	19	ITG, MTG, FG	1.45	2.57	.010	.036
Spanish-English Bilingual						
L	8	Precentral, Postcentral	2.23	4.33	<.001	<.001
L	22	MOG, MTG, ITG	3.66	8.20	<.001	<.001
R	6	IFG, Precentral, MFG	1.72	2.57	.010	.043

Table S5

Brain Activations during the Lexical Compound Condition Relative to the Derivational Affixes Condition

Hemisphere	Channel	Region	*β*	*t*-stat	*p*	*q*
English Monolingual						
R	4	dIFG, MFG	-1.09	-2.76	.005	.030
R	21	MOG, ITG, FG, MTG	-1.17	-2.51	.012	.047
R	22	MOG, MTG, ITG	-1.62	-2.84	.005	.026
Chinese-English Bilingual						
L	13	SMG, STG, MTG, IPL	-1.34	-3.42	<.001	.006
L	16	AG, Precuneus, IPL, STG	-1.74	-3.56	<.001	.004
L	23	ITG, IOG, MOG, FG	-3.52	-9.91	<.001	<.001
R	11	STG, SMG, IPL, Postcentral	-1.63	-2.67	.008	.030
R	21	MOG, ITG, FG, MTG	-1.62	-2.60	.009	.035
Spanish-English Bilingual						
L	2	MFG, dIFG	-2.03	-3.17	.002	.009
L	11	STG, SMG, IPL, Postcentral	-1.52	-2.71	.007	.030
L	19	ITG, MTG, FG	-1.37	-3.15	.002	.009
R	4	dIFG, MFG	-2.57	-5.15	<.001	<.001
R	13	SMG, STG, MTG, IPL	-1.08	-2.73	.006	.030
R	14	IPL, SMG, AG	-1.72	-3.36	<.001	.007
R	15	MTG, AG, STG, SMG	-2.38	-5.13	<.001	<.001

Table S6

Brain Activations Across Language Group by Morphological Task Condition

Hemisphere	Channel	Region	*β*	*t*-stat	*p*	*q*
Compound: Chinese Bilingual > Spanish Bilingual						
L	22	MOG, MTG, ITG	3.29	-3.85	<.001	.011
R	17	MTG, STG	-4.07	-3.85	<.001	.011
Derivational: Chinese Bilingual > Monolingual						
L	2	MFG, dIFG	3.48	3.93	<.001	.002
L	13	SMG, STG, MTG, IPL	-2.51	-3.23	.001	.019
L	16	AG, Precuneus, IPL, STG	-3.67	-4.31	<.001	<.001
L	22	MOG, MTG, ITG	2.76	3.03	.002	.033
L	23	ITG, IOG, MOG, FG	-3.43	-3.92	<.001	.002
Derivational: Spanish Bilingual > Monolingual						
L	22	MOG, MTG, ITG	8.10	4.89	<.001	<.001
R	4	dIFG, MFG	-4.51	-3.05	.002	.050
R	14	IPL, SMG, AG	-5.53	-3.39	<.001	.022
Derivational: Chinese Bilingual > Spanish Bilingual						
L	2	MOG, MTG, ITG	4.55	4.54	<.001	<.001
L	7	Postcentral, STG, Precentral	3.41	3.69	<.001	.007
L	15	MTG, AG, STG, SMG	-2.55	-3.27	.001	.020
R	4	dIFG, MFG	3.48	4.02	<.001	.003
